# Identification of novel non-coding RNAs using profiles of short sequence reads from next generation sequencing data

**DOI:** 10.1186/1471-2164-11-77

**Published:** 2010-02-01

**Authors:** Chol-Hee Jung, Martin A Hansen, Igor V Makunin, Darren J Korbie, John S Mattick

**Affiliations:** 1 Institute for Molecular Bioscience, University of Queensland, St Lucia QLD 4072, Australia; 2 Department of Molecular and Cellular Biology, Institute of Chemical Biology and Fundamental Medicine, 630090 Novosibirsk, Russia

## Abstract

**Background:**

The increasing interest in small non-coding RNAs (ncRNAs) such as microRNAs (miRNAs), small interfering RNAs (siRNAs) and Piwi-interacting RNAs (piRNAs) and recent advances in sequencing technology have yielded large numbers of short (18-32 nt) RNA sequences from different organisms, some of which are derived from small nucleolar RNAs (snoRNAs) and transfer RNAs (tRNAs). We observed that these short ncRNAs frequently cover the entire length of annotated snoRNAs or tRNAs, which suggests that other loci specifying similar ncRNAs can be identified by clusters of short RNA sequences.

**Results:**

We combined publicly available datasets of tens of millions of short RNA sequence tags from *Drosophila melanogaster*, and mapped them to the *Drosophila *genome. Approximately 6 million perfectly mapping sequence tags were then assembled into 521,302 tag-contigs (TCs) based on tag overlap. Most transposon-derived sequences, exons and annotated miRNAs, tRNAs and snoRNAs are detected by TCs, which show distinct patterns of length and tag-depth for different categories. The typical length and tag-depth of snoRNA-derived TCs was used to predict 7 previously unrecognized box H/ACA and 26 box C/D snoRNA candidates. We also identified one snRNA candidate and 86 loci with a high number of tags that are yet to be annotated, 7 of which have a particular 18mer motif and are located in introns of genes involved in development. A subset of new snoRNA candidates and putative ncRNA candidates was verified by Northern blot.

**Conclusions:**

In this study, we have introduced a new approach to identify new members of known classes of ncRNAs based on the features of TCs corresponding to known ncRNAs. A large number of the identified TCs are yet to be examined experimentally suggesting that many more novel ncRNAs remain to be discovered.

## Background

Following the discovery of microRNAs (miRNAs) and the RNA interference pathway in *C. elegans *[1], and the realisation that small RNAs are central to many aspects of plant and animal gene regulation, especially during development [2,3], have led to the identification of thousands of miRNAs in many species through deep sequencing-based approaches [4-6]. Such approaches have also identified related small ncRNAs including small interfering RNAs (siRNAs) and Piwi-interacting RNAs (piRNAs) that are involved in RNA-silencing pathways in somatic and germline cells, respectively [7-10]. Moreover, recent advances in sequencing technology have increased the understanding of biogenesis of these ncRNAs through deep-sequencing of size-fractionated RNA fragments associated with particular proteins [8,9,11-20], and also increased the amount of available short RNA sequencing data dramatically.

Analysis of such data has shown that miRNA-sized short RNA fragments are commonly derived from other small RNAs, notably transfer RNAs (tRNAs) and small nucleolar RNAs (snoRNAs) [21,22]. Indeed it is now evident that almost all snoRNAs produce defined classes of small RNAs that have characteristic sizes, origins within the snoRNAs, and 5' nucleotide biases [22], some of which may function as miRNAs [23]. In analyzing such data we observed that many annotated snoRNAs and other ncRNAs are in fact covered with overlapping short RNA tags across their full length, although most are derived from particular locations within the ncRNAs. A similar observation was also employed recently for computational prediction of novel snoRNAs in the Arabidopsis genome [24]. The observation of precursor coverage suggested to us that the profiles of overlapping short RNA sequences might identify novel members of known ncRNA classes and perhaps putative novel species of ncRNAs. In this study we confirm this prediction by using assembled short RNA sequences to identify one new snRNA, 7 new box H/ACA and 26 new box C/D snoRNAs, as well as a number of novel ncRNAs.

## Results

### Compilation of short RNA sequence reads into tag-contigs

**Figure 1:**
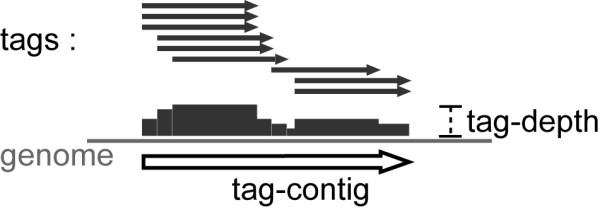
**Compilation of a tag-contig**. Contiguously overlapping tags (grey arrows) were assembled into a tag-contig (TC) (block arrow). The tag-depth is the maximum number of overlaying sequence reads per each base (grey blocks) within a given TC.

We obtained 10,846,433 sequence tags comprising 55,894,809 reads from 12 Gene Expression Omnibus (GEO) datasets (Table 1) derived from 90 experiments performed on *Drosophila *cell lines and tissues. Approximately 6 million tags were perfectly mapped to the *D. melanogaster *genome, excluding chrM (mitochondrial DNA), and chrU and chrUextra (which contain un-assembled and un-mapped scaffolds). Each "tag" consists of one to many reads which reflects the number of times the tag was cloned and sequenced. For tags mapping to multiple locations on the genome, the number of reads of the given tag was arbitrarily distributed evenly to each mapping locus (see Methods). The tags were then assembled into 521,302 tag-contigs (TCs), comprised of contiguously overlapping (by 1 nt or more) tags present in the same strand orientation (Fig. 1) (see Methods). As a measure of expression level, each TC was assigned a tag-depth score based on the maximum number of overlapping reads covering any part of the locus (Fig. 1) (see Methods).

**Table 1:** Publicly available short RNA sequencing datasets on *D. malanogaster*

GEO accession	No. of tags	Mappable	References
GSE10277	23252	12057	[14]

GSE10515	49878	12096	[15]

GSE10790	347861	30780	[19]

GSE10794	1387144	692422	[16]

GSE11019	255670	381508	[9]

GSE11086	1277025	1509771	[13]

GSE11624	6643474	3125323	[12]

GSE6734	32160	34362	[8]

GSE7448	753797	452471	[17]

GSE9138	13299	13294	[20]

GSE9389	59906	32472	[18,9]

GSE12527	2967	817	[11]

total	10846433	6297373	

The 521,302 TCs, ranging from 12 nt to 2734 nt in length and from 1 to 1767905 in tag-depth, occupy 14.6 Mbp (11.3%) of *Drosophila *genome in total (excluding chrU, chrUextra and chrM). Several studies have identified siRNAs and piRNAs processed from transposons such as LINEs, DNAs and LTRs, and correspondingly there is considerable TC coverage over these repeat elements (Additional file 1, Table S1). Most exons (75%) are also overlapped by TCs, possibly derived from messenger RNA (mRNA) (Additional file 1, Table S1). In addition, TCs identify 153 miRNAs out of 154 annotated miRNAs in the miRbase release 12.0 [25]. Interestingly, although all deep-sequencing data were size-fractionated to 18-32 nt, which encompasses the size of mature miRNAs, we found that some precursor miRNAs (pre-miRNAs) have most of their nucleotides covered by TCs (Table 2). This is also observed for other small ncRNAs such as snoRNAs and tRNAs. Most annotated box H/ACA and box C/D snoRNAs, tRNAs and small nuclear RNAs (snRNAs) in FlyBase [26] have overlapping TCs covering 70% or more of their length, and many are covered by single TCs that cover much of their length (Table 2). Moreover the majority of small non-messenger RNAs (snmRNAs) that have been experimentally validated as stable ncRNA transcripts with as yet uncharacterized functions [27] also have overlapping TCs (Table 2).

**Table 2:** Coverage of TCs over annotated ncRNAs

	annotated	overlapped by TCs (%)	≥ 70% coverage by TCs (%)	≥ 70% coverage by single TC (%)
mature miRNA	154	153 (99.3)	153 (99.3)	153 (99.3)

pre-miRNA	152	151 (99.3)	97 (63.8)	64 (42.1)

Box H/ACA snoRNAs	115	109 (94.8)	102 (88.7)	72 (62.6)

Box C/D snoRNAs	134	107 (79.9)	104 (77.6)	87 (64.9)

tRNAs	297	295 (99.3)	284 (95.6)	176 (59.3)

snRNAs	31	29 (93.5)	27 (87.1)	26 (83.9)

snmRNAs	20*	17 (85.0)	8 (40.0)	5 (25.0)

*Other 20 snmRNAs that are associated with *His *gene cluster were excluded due to their repetitive nature.

### TCs differ in their length and tag-depth for different classes of ncRNAs

**Figure 2:**
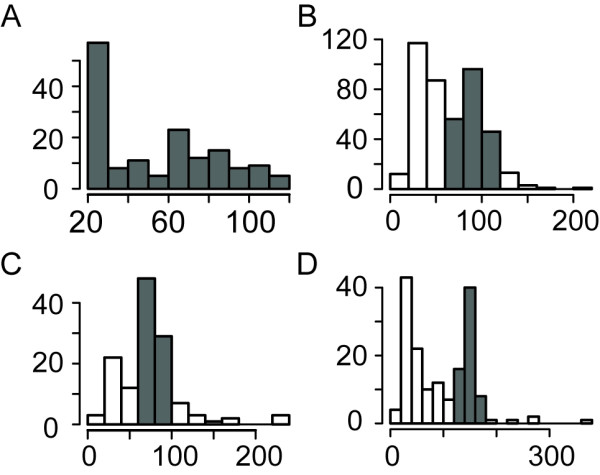
**Length distributions of TCs derived from different classes of ncRNAs**. (A) While many TCs overlapping with mature miRNAs are of 20-30 nt which is mature miRNAs' typical size range, many also span to the size of precursor miRNAs, typically up to 120 nt in *Drosophila *(grey bars). (B) Approximately a half of tRNA-derived TCs overlap full-length tRNAs, and some extend to the uncleaved 5' and/or 3' ends (grey bars). (C, D) Many TCs associated with box C/D and H/ACA snoRNAs are within the size ranges of their corresponding ncRNA classes (grey bars).

Most of the TCs that overlap with well-characterized classes of ncRNAs, such as miRNAs, tRNAs and snoRNAs, have particular length and tag-depth features that are consistent with the features of the corresponding ncRNA classes. TCs overlapping miRNAs show a high peak for the range of 20-30 nt in length (Fig. 2A), while TCs overlapping tRNAs and snoRNAs have bimodal length-distributions (Fig. 2B, C, D). TCs in the range of 60-120 nt overlap almost 70% of all *Drosophila *tRNAs. Some of these TCs are longer than the mature corresponding tRNAs, which in *Drosophila *fall within the range of 60-100 nt. This may well reflect the detection of uncleaved precursors [28], as these extended TCs had high tag-counts that may have more sensitively detected such precursors.

TCs covering the full length of 53% of all annotated box C/D snoRNAs and 56% of annotated box H/ACA snoRNAs (Fig. 2C, D) are within common size ranges of those two classes of snoRNAs (60-100 nt for box C/D snoRNAs and 120-180 nt for box H/ACA snoRNAs in *Drosophila*). The shorter TCs from tRNAs and snoRNAs that comprise the peaks on the left-hand side of Fig. 2B, C and 2D may indicate specifically processed short RNAs [21-23] (see Discussion).

**Figure 3:**
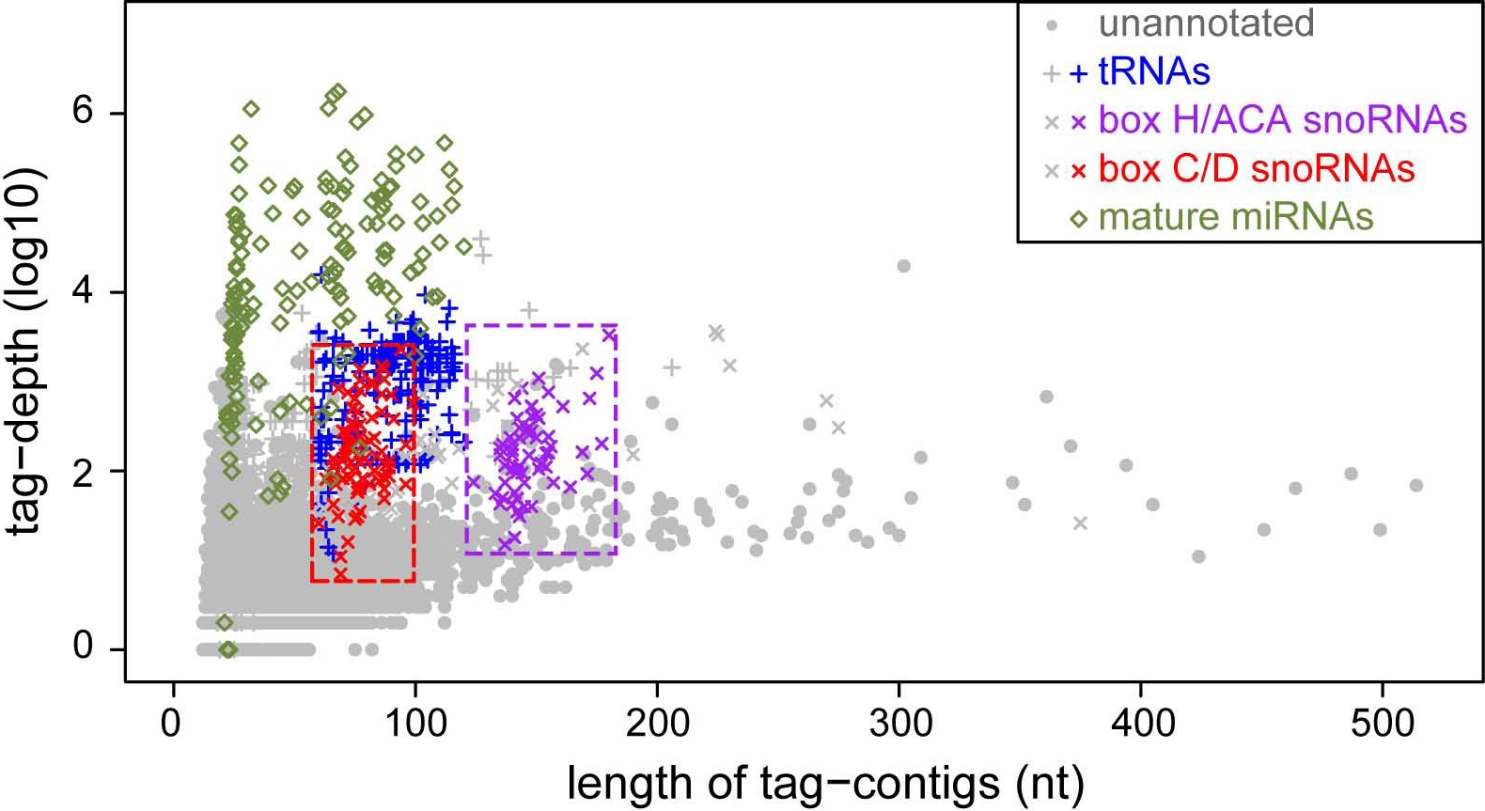
**The length and tag-depth distributions of TCs of different categories**. TCs overlapping 4 different classes of small ncRNA are plotted on top of all unannotated TCs. TCs comprising grey bars in Fig. 2 are coloured, and they differ in the length and tag-depth according to their corresponding ncRNA classes. While TCs derived from two different classes of snoRNAs have similar range of tag-depths, the TCs of box H/ACA snoRNAs (purple) are much longer than those of box C/D snoRNAs (red). tRNA-derived TCs (blue) overlap the size range with TCs of box C/D snoRNAs but tend to be deeper in tag-depth than box C/D snoRNA-derived TCs. TCs derived from miRNA loci are shown in green and show a wide range of tag-depths. Those spanning precursor miRNAs are scattered within the size ranges of box C/D snoRNAs or tRNAs, but have much greater tag-depths. The dashed boxes around the TCs for box C/D and box H/ACA snoRNAs show the areas within which box C/D and box H/ACA snoRNA candidates were searched for.

Fig. 3 adds another dimension to Fig. 2 by combining the tag-depth feature with the length feature of the TCs within the typical size ranges of corresponding ncRNAs (plotted on top of the unannotated TCs) and clearly discriminates different types of TCs derived from different classes of ncRNAs. TCs derived from miRNAs have a wide range of tag-depths (over 4 orders of magnitude) as many miRNAs are differentially expressed in different tissues or developmental stages [2,29]. TCs from the two classes of snoRNAs have similar ranges of tag-depths to each other, while the tag-depths of tRNA-derived TCs are generally higher than those of snoRNA-derived TCs. There are also many unannotated TCs within the size and tag-depth ranges of snoRNA-derived TCs (Fig. 3), indicating that some may also be unannotated snoRNAs, as well as other TCs that could potentially mark other unannotated ncRNAs. Hence we explored the predictive potential of TCs for the identification of new ncRNA loci.

### Filtering of unannotated TCs

For this analysis we excluded TCs derived from annotated exons, ncRNAs, transposons or other repeats annotated in FlyBase [26] (see Methods). While most of the remaining TCs do not overlap other TCs on the opposite strand, a substantial fraction (21%) of TCs have overlapping TCs on the other strand. The tag-depths of many of these are not particularly biased to either strand, causing ambiguity in transcription directions, which is characteristic of TCs derived from transposons (Additional file 1, Fig. S1) [9]. In contrast, TCs for known ncRNAs have either no overlapping TCs on the opposite strand or a strong bias in tag-depths towards the sense stand (Additional file 1, Fig. S1). Thus, we further excluded TCs that overlap other TCs on the opposite strands and do not show significantly greater tag-depths than the competing TCs (see Methods), and selected 100,193 TCs for further analysis.

### Prediction of seven novel box H/ACA snoRNAs and one snRNA

Among the 164 TCs overlapping known box H/ACA snoRNAs (Table 2), 64 are within the size range of 120-180 nt, covering full-length or near full-length of box H/ACA snoRNAs. The tag-depths of those 64 TCs ranged from 15 to 3,308 (Additional file 1, Fig. S2). We also observed that almost all annotated box H/ACA snoRNAs (106 out of 115) are located in annotated introns in the same transcriptional orientation as their host genes, as expected [30]. Thus, from the 100,193 unannotated TCs, we selected 20 TCs that are (i) intronic (sense), (ii) within size range of 120-180 nt and (iii) with tag-depth of at least 15. Subsequent motif analysis identified 9 of these TCs that have the characteristic box H (ANANNA) and box ACA motifs in the appropriate positions [31] (see Methods). BLAST analysis [32] revealed that one of those 9 TCs, although unannotated in FlyBase, had already been identified as a box H/ACA snoRNA (GenBank AJ809564) (Additional file 1, Table S2), providing a positive control for the analysis. It also showed that one TC at chr3R_1020733_1020883 had a sequence that is almost identical to a snRNA:U4atac:1 in *Drosophila simulans *(NCBI Reference Sequence XR_050942.1). Considering that most snRNAs in *D. simulans *were predicted from BLAST analysis with known snRNAs of *D. melanogaster *against the *D. simulans *genome sequence [33], the TC at chr3R_1020733_1020883 is also a candidate snRNA (Additional file 2). The remaining 7 TCs were classified as novel box H/ACA snoRNA candidates (Additional file 2). Interestingly, the box H/ACA snoRNA candidate TCs tend to have higher tag-depth compared to the 11 TCs excluded due to the absence of motifs. The average and median tag-depths of the 7 candidates TCs are 304 and 105, respectively, while those of excluded TCs are 188 and 44, respectively.

### Prediction of 26 box C/D snoRNAs

A total of 107 box C/D snoRNAs (out of 134 annotated in FlyBase [26]) are overlapped by 130 TCs (Table 2), of which 78 are within the typical size range of box C/D snoRNAs (60-100 nt). The tag-depth of these TCs ranged from 6 to 2,293 (Additional file 1, Fig. S2). Since box C/D snoRNAs are located either in introns (sense to introns) or in intergenic spacers, we selected those TCs from the 100,193 unannotated TCs that are (i) either intronic or intergenic, (ii) within the size range of 60-100 nt and (iii) with a tag-depth of at least 6. Out of 573 TCs that fulfilled these conditions, we found that 27 have the characteristic box C (RUGAUGA) and box D (CUGA) motifs in the vicinity of their 5' and 3' ends, respectively [31] (see Methods). One of these TCs, at chr3LHet_2398490_2398558, has been previously identified as a snoRNA (GenBank AJ784386) (Additional file 1, Table S2), again providing a positive control for the analysis. The remaining 26 TCs were considered candidate box C/D snoRNAs (Additional file 2). The average tag-depths of these candidate TCs is also much higher than those that do not have recognizable box C/D motifs - 71 and 23 for candidate TCs and excluded TCs, respectively, although the median tag-depths of those two TC sets are not significantly different from each other - 9 for both. BLAST analysis also showed that one candidate snoCD_05 (chr2L_6917229_6917303) is highly homologous to a box C/D snoRNA, snord53 (GenBank X96652.1), in human and mouse [34] (Additional file 1, Table S2), which subsequently also showed positive in Northern blots (see below).

### TCs for putative ncRNAs

After these box H/ACA and box C/D snoRNA predictions, 100,157 TCs still remain unannotated. To explore these further, we initially selected 135 highly expressed TCs with a tag-depth ≥100 as Fig. 3 shows that a large portion of TCs mapped to known ncRNAs have tag-depths of 100 or higher. BLAST analysis further excluded 49 TCs that have sequences homologous to annotated transposons. The remaining 86 were classified into three groups based on their length and tag-depth: 8 TCs that are shorter than 40 nt with tag depths of 1000 or more (group 1); 29 TCs that are shorter than 40 nt with tag depths of 100-999 (group 2); and the 49 longer TCs (group 3) (Additional file 1, Fig. S3 and Additional file 3).

The location of group 1 TCs is strongly biased to introns of genes involved in development (Table 3) and with the exception of ncRNA_05, at chrX_3721726_3721755, which is located immediately downstream of a tRNA-Pro locus, all share a consensus 18 nt sequence (18mer motif), GTCCACCCGGGGGCGCCA, which is also by far the most abundant sequence read within these TCs (31,689 out of 40,129 reads). Genomic scanning for the 18mer motif sites found 1 more exact match antisense to the 3' UTR of a gene of uncharacterized function (CG31665). Allowing 1 and 2 mismatches for the genomic scanning identified a further 17 and 31 sites, respectively (excluding chrU, chrUextra and chrM), the majority of which are also intronic (Additional file 1, Table S3). One of the 17 motif sites with 1 mismatch and 9 of the 31 motif sites with 2 mismatches were found at the 3'end of tRNA:N5 (Additional file 1, Table S3) which indicates that this motif may have arisen from tRNA:N5. However, none of the sequence tags contributing to the 7 TCs is mapped to the tRNA:N5, suggesting that these sequence tags are produced independently of tRNAs. The 7 Group1 TCs sharing the 18mer motif show high expression levels in S2 and Kc cell lines, while the tRNA-associated TC has a significantly high number of sequence reads from the mid-embryonic stages (Additional file 1, Fig. S4).

**Table 3:** TCs in Group1, unannotated but highly expressed TCs.

	locus	tag-depth	strand	TC size (nt)	Gene
ncRNA_01	chr2R_4733783_4733804	5027	+	21	*sns*

ncRNA_02	chr2R_9632216_9632238	6051	-	22	*fas*

ncRNA_03	chr2R_13693470_13693490	5460	-	20	*grh*

ncRNA_04	chr2R_19535102_19535122	5460	-	20	*retn*

ncRNA_06	chrX_11524384_11524406	5685	+	22	*Ptp10D*

ncRNA_07	chrX_12399632_12399653	5697	-	21	*CG2556*

ncRNA_08	chrX_19880356_19880381	6749	+	25	N/A

ncRNA_05*	chrX_3721726_3721755*	4733	+	29	*ec*

*Located downstream of a tRNA.

The 29 TCs in Group2 are evenly distributed in introns (13 TCs) and intergenic spacers (16 TCs), and intronic TCs and intergenic TCs do not differ in tag-depth distributions. Two of these TCs, ncRNA_12 and ncRNA_37, were detected by genomic scanning for the 18mer motif derived from the Group1 TCs when 1 mismatch was allowed. Those two TCs along with ncRNA_15 are mainly composed of sequence reads specifically obtained from S2 cells [35] (Additional file 1, Fig. S5). Two other TCs, ncRNA_20 and ncRNA_28, are composed of sequence reads heavily biased to adult body, larvae, and pupae (Additional file 1, Fig. S5).

In Group3, 3 of the 49 TCs were found to be derived from annotated ncRNAs by BLAST analysis (Additional file 1, Table S4). Those at chr2R_7292203_7292273 (nRNA_48) and chr2R_7292691_7292839 (ncRNA_49) have recently been annotated as tRNAs-Thr in the Genomic tRNAdb [36] and that at chr3R_2645849_2646151 (ncRNA_60) defines 7SL RNA precisely [37], further demonstrating that this approach is able to detect various types of small ncRNAs (Additional file 1, Table S4). We also found that a cluster of 17 150 nt-long TCs located within a 4.6 kb region, chrX:4,815,890-4,820,490, is part of an endogenous siRNA cluster identified by Czech *et al. *[13] (Additional file 1, Table S4). Each of the TCs in this cluster is an exact copy of the others and forms a hairpin structure which is the precursor of siRNAs. The remaining 29 TCs are not particularly enriched in introns, but intronic TCs tend to have higher tag-depths than intergenic TCs (Additional file 1, Table S5).

### Experimental validation of putative ncRNAs

**Figure 4:**
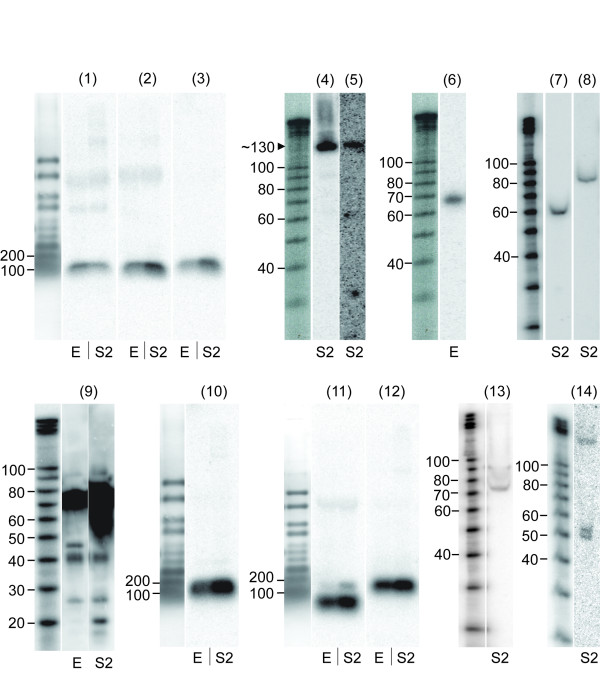
**Validation of selected ncRNA candidates**. Positive results of snRNA candidate: (1), selected box H/ACA snoRNA candidates: (2)-(5), box C/D snoRNA candidates: (6)-(8), 18mer motif for group1 TCs: (9) and selected group3 TCs: (10)-(14) (E: embryos of 12-18 h; S2: S2 cells). 2% agarose/formaldehyde gels with 25 micrograms of RNA from *D. melanogaster*'s embryo and S2 cells were used for (1), (2), (3), (10), (11) and (12), and 10% denaturing polyacrylamide gels with 25 micrograms of RNA from either embryos or S2 cells were used for the rest. The estimated sizes of bands are listed in Table 4. The left-most lanes of each of groups show the size standards. The estimated size of ~130 nt for the positive bands in lanes (4) and (5) is shown by the arrowhead. (1): snRNA_01; (2):snoHACA_01; (3):snoHACA_05; (4):snoHACA_04; (5): snoHACA_06; (6):snoCD_05; (7):snoCD_24; (8):snoCD_02; (9):ncRNA_01; (10):ncRNA_38; (11):ncRNA_39; (12):ncRNA_50; (13):ncRNA_47; (14):ncRNA_54.

For experimental validation of snRNA and snoRNA predictions, we selected the snRNA candidate along with top 4 and 5 box H/ACA and box C/D snoRNA candidates, respectively, based on the tag-depth. The snRNA candidate, all 4 box H/ACA snoRNA candidates and 3 of the box C/D snoRNA candidates tested positive by Northern blot (Table 4) (Fig. 4A, B), with clear bands of the approximately expected sizes based on the length of TCs. The 2 candidates that were not experimentally confirmed have relatively low tag-depths (27 each) compared to the others (at least 42), suggesting sensitivity problems and/or that the snoRNAs are not expressed in the cell line or embryonic stages tested (see Methods). In fact, while all confirmed snoRNA candidates have large number of sequence reads from late embryo (12-18 h) and S2 cells (Additional file 1, Fig. S6A, B) on which the experimental validations were performed (see Methods), the two unconfirmed box C/D snoRNA candidates have the highest number of sequence reads from early embryo (Additional file 1, Fig. S6B).

**Table 4:** Experimental validation results for selected novel ncRNA candidates.

	Type	tag-depth	TC size (nt)	Estimated size from Northern blot (nt)	Gene
snoHACA_01	box H/ACA snoRNA	105	145	~ 150	*SC35*

snoHACA_04	box H/ACA snoRNA	234	143	~ 130	*Dek*

snoHACA_05	box H/ACA snoRNA	106	137	~ 170	*hts*

snoHACA_06	box H/ACA snoRNA	1555	158	~ 130	*CG31191*

snRNA_01	snRNA:U4atac	282	150	~ 150	*cno*

					

snoCD_01	box C/D snoRNA	27	74	N/A	N/A

snoCD_02	box C/D snoRNA	42	81	~ 85	*kis*

snoCD_05	box C/D snoRNA	566	74	~ 70	*x16/nop*

snoCD_09	box C/D snoRNA	27	80	N/A	N/A

snoCD_24	box C/D snoRNA	993	69	~ 59	N/A

					

ncRNA_01*	Group1	5027	20~ 25^†^	18/21/26	^‡^

					

ncRNA_38	Group3 (intron)	1418	159	~ 150	*v(2)k05816*

ncRNA_39	Group3 (intron)	582	198	~ 90/~ 190	*D*

ncRNA_47	Group3 (intron)	418	124	~ 70	*gc1*

ncRNA_54	Group3 (intron)	153	93	~ 49/~ 55	*Stet*

ncRNA_64	Group3 (intron)	223	48	~ 70	*CG11882*

ncRNA_85	Group3 (intron)	225	72	N/A	*CG1718*

					

ncRNA_50	Group3 (intergenic)	106	172	~ 180	N/A

ncRNA_55	Group3 (intergenic)	180	79	N/A	N/A

ncRNA_62	Group3 (intergenic)	123	56	N/A	N/A

ncRNA_83	Group3 (intergenic)	116	394	N/A	N/A

*One of the 7 TCs in Group1 that shares an 18 nt long sequence. ^†^ncRNA_05 which is immediately downstream of a tRNA was excluded. ^‡^Introns of 6 genes shown in Table 3.

We also carried out Northern blots using the 18mer motif that dominates the tag spectrum in 7 out of 8 Group1 TCs, and observed a strongly hybridizing band of approximate size 21 nt along with weaker 18 nt and 26 nt bands in S2 cells with a different distribution in late-embryo (Fig. 4C). We also observed larger 42 nt, 48 nt and 79 nt bands, the latter of which (and perhaps others) may well be the result of cross-hybridization to highly abundant RNA molecules such as tRNAs, given the extremely high GC content of the 18mer motif (83.3%) and the high similarity to tRNA:N5 (Additional file 1, Table S3). In any case, the bands between 18 and 26 nt clearly suggest that the 18mer motif is expressed as small RNAs, and is consistent with the incidence and size of the tags covering this motif.

A subset of group 3 TCs was also tested by Northern blot. As the majority of TCs for known ncRNAs overlap phastCons elements [38] (Additional file 1, Table S6) (see Methods), we selected 6 intronic and 4 intergenic TCs that mapped to phastCons elements, 5 and 1 of which, respectively, showed positive in Northern blots (Table 4) (Fig. 4D). Two exhibited clear bands of the approximately expected sizes based on the TC lengths, whereas the other four exhibited bands that are either shorter or longer than the lengths of their corresponding TCs (Table 4). For the three TCs with shorter sized bands, ncRNA_47 (chr2R_4612441_4612565), ncRNA_54 (chr3L_1488738_1488831) and ncRNA_39 (chr2L_8485729_8485927), the number of overlaying reads is very low in a few parts of each, suggesting that those TCs may represent unprocessed precursors (Additional file 1, Fig. S7). On the other hand, an intronic TC ncRNA_64 (chr3R_24973624_24973672) showed a very weak band of approximately 70 bp (data not shown), while its expected length was 48 nt (Table 4). Considering that this TC is located within a 70 nt long intron of CG11882, it may be that the actual transcript detected by Northern covers the entire intron (Additional file 1, Fig. S8). The numbers of sequence reads for TCs with weak signals, ncRNA_54 (chr3L_1488738_1488831) (Fig. 4D) and ncRNA_64 (chr3R_24973624_24973672) (data not shown), were lower than those of the other confirmed TCs, and the most reads for those two TCs were obtained from mid-embryonic stages (Additional file 1, Fig. S6C). This could be the reason for the weak signals from Northern blot as is for the unconfirmed box C/D snoRNA candidates. The 4 unconfirmed TCs also have small number of sequence reads from late embryos and S2 cells, while most of their sequence reads are from early and mid-embryonic stages (Additional file 1, Fig. S6C).

Probe sequences for the Northern blotting analysis are provided in Additional file 4, and the sequences of validated ncRNA candidates are provided in FASTA format in Additional file 5.

## Discussion

In this study, we utilized a strategy of analyzing millions of short reads from next generation sequencing experiments for the prediction of novel ncRNAs of both known and unknown classes. Although the deep-sequencing analyses used in this study focus on identifying shorter ncRNAs such as miRNAs, siRNAs and piRNAs by limiting the lengths of the RNA samples to the sizes of such small ncRNAs, assemblages of contiguously overlapping tags also overlap with longer ncRNAs such as snoRNAs, snRNAs and tRNAs.

TCs derived from two different classes of snoRNAs showed distinct features in their length and tag-depth distributions, and the use of these characteristic features along with the their signature motifs predicted novel snoRNAs. Proof-of-principle of this approach is provided by the successful recall of two previously known but not FlyBase-annotated snoRNAs as well as the *de novo *identification of three known ncRNAs (two tRNAs and 7SL RNA) and an endogenous-siRNA cluster. We also found that the majority of experimentally detected snmRNAs [27] (excluding those that are related to *His *clusters) are overlapped by TCs, another demonstration of the validity of the approach. In fact, one TC (chr3R_3300274_3300719) overlapping *snmRNA:331 *corresponds to the 7SK RNA recently identified in *Drosophila *[39], the boundaries of which fit better to the 5' and 3' ends of the TC than those of *snmRNA:331*.

Characteristic features of snoRNAs were extracted from TCs that cover the full-length of annotated snoRNAs. However, there are also many short TCs partially overlapping with annotated snoRNAs, with strong positional preference in both 5'/3' ends of snoRNAs, which is consistent with the positional preferences of snoRNA-derived small RNAs (sdRNAs) [22]. These positional preferences were also observed and used for novel snoRNA predictions in the Arabidopsis genome [24]. We also found that these short TCs within snoRNAs were closely juxtaposed. Thus, more accumulation of deep-sequencing data would be expected to connect these TCs and identify more novel snoRNAs. We also examined the potential of making a simple merge of closely located TCs but this approach was compromised by also merging adjacent snoRNAs. Chen *et al*. [24] bypassed this problem in their snoRNA predictions by first anchoring the 3' ends of the novel snoRNA transcripts and then looking for their 5' ends. However, this method cannot be easily generalized for ncRNAs of uncharacterized classes. Alternatively, carefully designed computational approaches using the distribution of short RNA tags across annotated snoRNAs may also increase the number of novel snoRNAs predictions. Our candidates were tested by the snoRNA prediction software *SnoReport*, which also refuses to use the modification target information of snoRNAs [40], but it identified (using the default options) only 3 box H/ACA and 5 box C/D snoRNAs from our 7 and 26 snoRNA candidates, respectively. However, when we tested the performance of *SnoReport *on the 115 box H/ACA and 134 box C/D snoRNAs that are annotated in the *Drosophila melanogaster *genome, only 59 box H/ACA and 51 box C/D snoRNAs were successfully recalled.

Unlike the prediction of box C/D snoRNAs and putative ncRNAs of uncharacterized classes, the box H/ACA snoRNA prediction incorporated another filter that excluded non-intronic TCs. This was based on the fact that 92% of the known box H/ACA snoRNAs reside in introns, and reduced the number of predictions from 18 (based on tag-contig size, tag depth and presence of the H/ACA motif) to 7. It is uncertain how many of the 10 discarded TCs (excluding one snRNA candidate) may be genuine box H/ACA snoRNAs, but the high validation rate of the intronic subset (4 out of 4 tested) indicates that the incorporation of the location filter improved the specificity of the prediction.

The length and tag-depth distributions of unannotated TCs are similar to those of exon-derived TCs (Additional file 1, Fig. S9A, B), which may indicate that some unannotated TCs might be assemblages of degradation products of unknown exons. However, it is equally possible that they may also represent degraded or processed fragments of *bona fide *ncRNAs that can also be re-assembled, as is evidently the case for snoRNAs. Moreover, the large amount of unannotated TCs located in introns and intergenic regions (Additional file 1, Fig. S9C, D) indicates that there are many more unknown transcripts yet to be investigated. Considering that we used a conservative threshold of tag-depths (≥100) for uncharacterised ncRNA candidates as the vast majority of exon-derived TCs (99.9%) have tag-depths less than 100, the novel ncRNA candidates shown in this study are just the tip of the iceberg. We tested 10 of the 29 putative ncRNA candidates in group 3, focusing on those that were most highly conserved, 6 of which returned positive signals in a restricted range of cells (see below). However, considering that some ncRNAs evolve at high rate [41], the untested 19 ncRNA candidates in group 3 could equally likely be novel ncRNAs. Indeed, among the total of 100,193 unannotated TCs, only 26,395 overlap phastCons elements, and, surprisingly, there is no apparent difference in the distributions of lengths and tag-depths between TCs that overlap conserved sequences and those do not (Additional file 1, Fig. S9E, F). This suggests that while conservation may be used as a positive guide to likely ncRNAs, the relative lack of conservation is not necessarily an index of lack of relevance of others.

**Figure 5:**
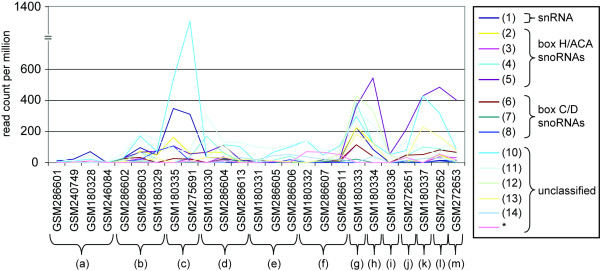
**Number of sequences per million from each experiment for confirmed TCs**. (a) heads, (b) adult body, (c) imaginal discs, (d) very early embryo (0-1 h), (e) early embryo (2-6 h), (f) mid embryo (6-10 h), (g) late embryo (12-24 h), (h) larvae: 1st instar and 3rd instars, (i) pupae: 0-1 day, 0-2 day, 2-4 day, (j) S2 and KC cells, (k) tissue culture cells (S2 only), (l) S2 cells, (m) KC cells. Numbers for TCs shown on right panel are correspondent to those in Fig. 4 (*: not shown in Fig. 4). Overall they show similar expression profiles in different tissues of developmental stages. Expression profiles of Group1 TCs including ncRNA_01 are shown in Additional file 1, Fig. S4. Expression profiles of all tested TCs are shown in Additional file 1, Fig. S5.

In the experimental validation, 15 out of 21 selected candidate TCs were positive - 8 out of 10 selected snRNA and snoRNA candidates (80%) and 7 of 11 (64%) putative unclassified transcripts of either group 1 or group 3. This is a high rate of validation, given that the likelihood of detecting a signal in Northern blots is dependent on the expression level of the candidate in the tissue concerned. In fact, the confirmed candidates have generally greater tag-depths than the unconfirmed (Table 4), and they are also more contributed by tags obtained from late embryos or S2 cells (Fig. 5) that were the source material for Northern blots. In contrast, among the confirmed candidates, the TCs with weaker signals have a lower number of sequence reads from late embryonic stages than other confirmed candidates (Fig. 5).

In addition, there remain a large set of ~ 27,000 TCs that overlap TCs on complementary strands, which is characteristic of TCs mapping to transposons. They are also closely located to each other (≤100 bp), similar to TCs covering known transposon-derived sequences, and different to the ~ 100,000 TCs which were used for this study. We also observed that a large portion (37%) of these 27,000 TCs is found within reported siRNA/piRNA clusters [8,9,13,14,20,42]. Although some of siRNA or piRNA clusters are not associated with transposons [13], these preliminary observations indicate that some of these complementary TCs may be derived from unidentified transposons. In fact, about five thousand TCs in this set either slightly overlap with or are located close to (≤100 bp) existing transposons have sequences homologous to transposons, suggesting they could be unannotated parts of existing transposons generating siRNAs or piRNAs.

## Conclusions

Several studies investigating the population of small RNAs have yielded millions of sequence reads. In this study, we combined all publicly available sequence data from *Drosophila melanogaster *short RNA into hundreds of thousands tag-contigs and associated subsets of them with known ncRNAs such as snoRNAs and tRNAs. The characteristic features of TCs overlapping with known ncRNAs were used to predict 7 and 26 box H/ACA and box C/D snoRNA candidates, respectively, in addition to one snRNA and many novel unclassified ncRNA candidates, a substantial fraction of which were experimentally validated. We conclude that deep sequencing from short reads may be used to identify new members of known and novel classes of ncRNAs, including those that are significantly longer than the reads themselves.

## Methods

### Genome sequence and annotation

We used the *D. melanogaster *genome sequence assembly Release 3 (April, 2006) from the Berkeley *Drosophila *Genome Project. Annotation of exons, introns, UTRs, and ncRNAs are from FlyBase 5.12 [26]. MicroRNA annotation was obtained from miRbase release 12.0 [25]. Repeats were annotated using RepeatMasker [43] in FlyBase 5.12.

### Mapping of sequence tags

We obtained all public available deep-sequencing datasets from Gene Expression Omnibus database at National Center for Biotechnology Information http://www.ncbi.nlm.nih.gov/geo in SOFT format (Table 1). These sequences were subsequently mapped to the genome of *D. melanogaster *using Vmatch http://www.vmatch.de and a bioinformatics toolkit - Biopieces http://www.biopieces.org, to obtain all full length exact hits. Hits on chrM, chrU and chrUextra were discarded. Each tag in the dataset is comprised of a number of reads, i.e., the number of times the tag was sequenced. For tags mapping to unique locations in the genome, it is obvious that the mapped locus was cloned and sequenced as many times as the number of reads of the given tag. For tags mapping to multiple loci, the number of reads of the given tag was distributed evenly to each mapped locus, and transcripts from each locus were assumed to have been cloned and sequenced as many times as the number of reads of the tag divided by the number of mapped loci [8]. Sequence tags that had a greater number of mapped loci than the number of reads were discarded.

### Tag contigs

A tag-contig (TC) is defined as a genomic region that has mapped sequence tags with the same strand orientation contiguously overlapping with each other by at least 1 nt (Fig. 1). Each base within a TC is overlayed by the number of reads that include the base (adjusted number of reads for multi-mapping tags), and the maximum accumulation of read numbers within a given TC is defined as the tag-depth for the TC (Fig. 1).

### Classification of TCs

TCs overlapping at least 20% of the length with exons, introns, repeats and annotated ncRNAs were classified as TCs derived from each of the annotations. TCs have less than 20% overlap with exons, genic regions and repeats were regarded as non-exonic (or intronic), intergenic and non-repeat TCs, respectively. Intersection of TCs with other annotation was performed through local mirror of University of California, Santa Cruz Genome Browser [44].

### Selection of TCs for analysis

Among the total of 521,302 TCs, 126,962 are outside of annotated exons, ncRNAs, transposons and other repeats annotated in FlyBase [26]. Of these, 27,151 TCs overlap with other TCs mapped to the complementary strand. Most TCs sense to known ncRNAs have at least 10 times greater tag-depth than those that are antisense to known ncRNAs (Additional file 1, Fig. S1). Based on this observation of fold-differences, we selected 382 from the ~ 27,000 TCs, that have at least 10 times greater tag-depth than their overlapping TCs on the opposite strand.

### Scatter plotting of TCs

The scatter-plots of lengths (nt) against tag-depth (log10) of TCs were generated by R http://www.R-project.org and in-house software along with the bioinformatics toolkit, Biopieces http://www.biopieces.org.

### Conservation of sequence reads

A sequence tag overlapping with phastCons elements [38] by at least 15 bp is considered as a conserved sequence tag. Each sequence tag represents a number of sequence reads, thus sequence reads comprising the conserved tags are also regarded as conserved reads.

### Motifs in snoRNA candidate TCs

For each of the unannotated TCs within the ranges of length and tag-depth of box C/D snoRNA-derived TCs, box C motif (RUGAUGA) and box D motif (CUGA) were searched within +/- 10 bp from the 5' end and within +/- 10 bp from the 3' end, respectively [40]. One mismatch was allowed for both box C and D motifs. For the box H/ACA snoRNA predictions, 20 bp of flanking sequences of the midpoint of a TC were searched for the box H motif (ANANNA), and 20 bp of flanking sequence of 3' end of a TC were examined for the box ACA motif [40].

### Gene Ontology analysis

The Gene Ontology term enrichment analyses in this study were performed using GO-TermFinder [45] through the AmiGO web site http://amigo.geneontology.org/cgi-bin/amigo/term_enrichment.

### Northern blots

Total RNA was extracted from *Drosophila*'s late embryos (12-18 h) and S2 cells using TRIZOL reagent (Invitrogen). Fifteen micrograms of total RNA was separated on 1% denaturing agarose gels, and 25 micrograms of total RNA was on 2% agarose/formaldehyde gels and 10% denaturing polyacrylamide gels. RNA separated using denaturing agarose was then transferred to Hybond-N+ membranes (GE Healthcare) using downward capillary transfer, then UV-crosslinked and baked at 80°C for 1 hour. RNA separated by denaturing polyacrylamide gels was transferred to Hybond-Nx membranes (GE Healthcare) by use of a semidry transfer cell apparatus, and cross-linked using the EDC method as outlined in [46]. Antisense oligonucleotides complementary to predicted ncRNA candidates were used as probes. Northern blotting was carried out as described by Nelson Lau from Bartel Laboratory, http://web.wi.mit.edu/bartel/pub/protocols/miRNA_Nrthrns_Protocol.pdf. In brief, the pre-hybridization/hybridization buffer contained 5× SSC, 20 mM Na2HPO4 pH 7.2, 7% SDS, and 2× Denhardt's solution. Blots were pre-hybridized for at least 2 hours at 50°C, then probes which had been end-labeled with γ-32P ATP by use of T4 polynucleotide kinase (New England Biolabs), or end-labeled with α-32P dCTP by use of terminal transferase (New England Biolabs), were added to the hybridization chamber and incubated with the blots overnight at 50°C. After three washes with non-stringent wash buffer containing 3× SSC, 25 mM NaH2PO4 pH 7.5, and 5% SDS, blots were given a final wash with 1× SSC and 1% SDS. The membrane was then exposed to a phosphoimager overnight and scanned.

### Secondary structure analysis

The secondary structures of 7 box H/ACA snoRNA candidates (Additional file 6) were predicted by RNAfold [47]. In the case of snoHACA_07 (at chrX_915376_915513), the 5' and 3' ends were extended by 10 bp to include the box ACA motif.

## Authors' contributions

CJ contributed to the study design, carried out the data analysis and drafted the manuscript. MAH contributed to the study design and carried out the data collection and preparation. IVM contributed to the study design and helped draft the manuscript. DJK carried out the experimental validations. JSM supervised the study and helped write the manuscript. All authors read and approved the final manuscript.

## Authors' Information

IVM current address: Department of Molecular and Cellular Biology, Institute of Chemical Biology and Fundamental Medicine, 630090 Novosibirsk, Russia.

## Supplementary Material

Additional file 1 **Supplementary figures and tables**. This file contains supplementary figures S1-S9 and tables S1-S6 in PDF format. Fig. S1: The distributions of relative tag-depths of TCs against oppositely stranded overlapping TCs; Fig. S2: Tag-depth distributions of TCs covering full-length ncRNAs; Fig. S3: Length and tag-depth distribution of highly expressed unannotated TCs; Fig. S4: Number of sequence reads per million per different samples for Group1 TCs; Fig. S5: Number of sequence reads per million per different samples for Group2 TCs; Fig. S6: Number of sequence reads per million per sample for each of tested snoRNA and unclassified ncRNA candidates; Fig. S7: The accumulation of sequence reads per each base across the three TCs; Fig. S8: Screenshot of UCSC genome browser for ncRNA_64, an intronic TC at chr3R_24973624_24973672; Fig. S9: Length and tag-depth distributions of TCs of different categories; Table S1: Coverage of TCs over transposons and exons; Table S2: Predictions of snoRNA candidates; Table S3: 18mer motif sites derived from group1 TCs with 0, 1 and 2 mismatches; Table S4: Group3 TCs mapped to existing ncRNAs; Table S5: 29 unannotated TCs in group3; Table S6: Conservation of sequence tags comprising ncRNA-derived TCs. Click here for file

Additional file 2 **List of all snRNA, box C/D and box H/ACA snoRNA candidates**. This file contains genomic loci, strand, tag-depths and predicted lengths of 7 box H/ACA snoRNA candidates, 26 box C/D snoRNA candidates and 1 snRNA candidate. Click here for file

Additional file 3 **List of all group1, group2 and group3 TCs**. This file contains genomic loci, strand, tag-depths and predicted lengths of all putative ncRNAs of uncharacterized classes: 8 group1 ncRNAs, 29 group2 ncRNAs and 49 group3 ncRNAs. Click here for file

Additional file 4 **List of probes for Northern blot**. This file contains probe sequences for experimental validation of 4 box H/ACA snoRNA candidates, 5 box C/D snoRNA candidates, 1 snRNA candidate and 10 putative ncRNA of uncharacterized class along with their other information such as genomic loci, predicted lengths and tag-depths. Click here for file

Additional file 5 **Sequences of experimentally validated novel snoRNAs and unclassified ncRNA candidates**. This file contains DNA sequences of all experimentally validated ncRNAs: 4 box H/ACA snoRNAs, 3 box C/D snoRNAs, 1 snRNA and 13 ncRNAs of uncharacterized class. Click here for file

Additional file 6 **Sequence conservation and secondary structure predictions of snoRNA candidates**. This file contains UCSC genome browser screenshots of 7 box H/ACA snoRNA candidates and 26 box C/D snoRNA candidates showing sequence conservation and tag distributions over each candidate. The positions of box H, ACA, C and D motifs are indicated by the red boxes. Predicted secondary structures of box H/ACA snoRNA candidates are provided below each of the screenshots. Click here for file
