# Supplementary material for: Identification of novel non-coding RNAs using profiles of short sequence reads from next generation sequencing data
Source: BMC Genomics. 2010 Feb 1;11:77. doi: 10.1186/1471-2164-11-77 (PMC2825236; doi:10.1186/1471-2164-11-77)
Supplement: Additional file 1 — Supplementary figures and tables. This file contains supplementary figures S1-S9 and tables S1-S6 in PDF format. Fig. S1: The distributions of relative tag-depths of TCs against oppositely stranded overlapping TCs; Fig. S2: Tag-depth distributions of TCs covering full-length ncRNAs; Fig. S3: Length and tag-depth distribution of highly expressed unannotated TCs; Fig. S4: Number of sequence reads per million per different samples for Group1 TCs; Fig. S5: Number of sequence reads per million per different samples for Group2 TCs; Fig. S6: Number of sequence reads per million per sample for each of tested snoRNA and unclassified ncRNA candidates; Fig. S7: The accumulation of sequence reads per each base across the three TCs; Fig. S8: Screenshot of UCSC genome browser for ncRNA_64, an intronic TC at chr3R_24973624_24973672; Fig. S9: Length and tag-depth distributions of TCs of different categories; Table S1: Coverage of TCs over transposons and exons; Table S2: Predictions of snoRNA candidates; Table S3: 18mer motif sites derived from group1 TCs with 0, 1 and 2 mismatches; Table S4: Group3 TCs mapped to existing ncRNAs; Table S5: 29 unannotated TCs in group3; Table S6: Conservation of sequence tags comprising ncRNA-derived TCs. [file 1471-2164-11-77-S1.PDF]

Identification of novel non-coding RNAs using profiles of short sequence reads from next generation sequencing data

Jung et al.

Supplementary Figures S1-S9 and Supplementary Tables S1-S6

Figure S1

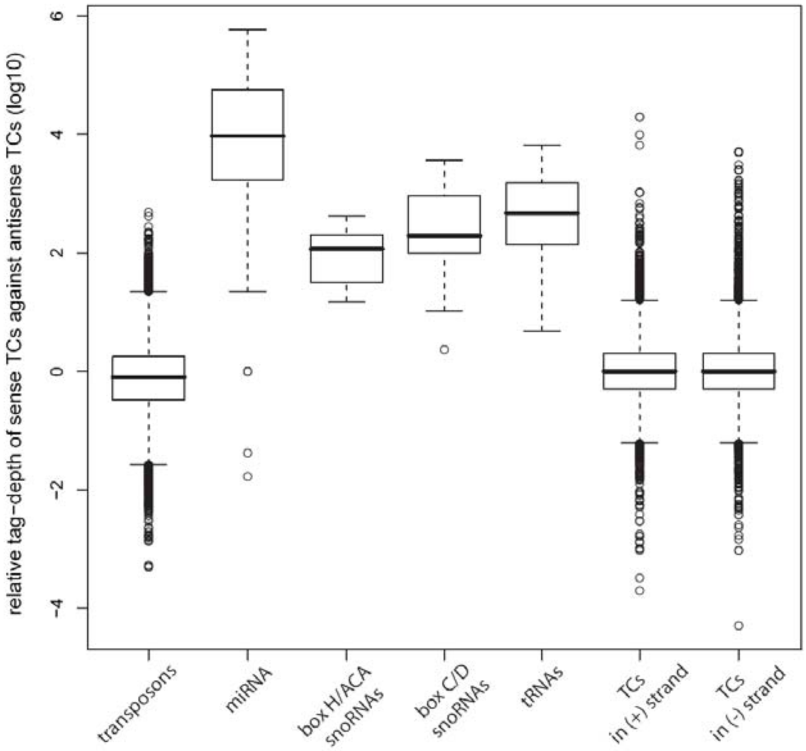

Figure S1. The distributions of relative tag-depths of TCs against oppositely stranded overlapping TCs. TCs with same strand orientations to the overlapping ncRNAs have higher tag-depths than oppositely stranded TCs against ncRNAs, while repeat-associated TCs are not particularly biased to the strands of the overlapping repeats. The relative tag-depth distributions of TCs overlapping with other TCs in the opposite strands are similar to those of transposon-associated TCs, suggesting some of them could be derived from unannotated transposons.

Figure S2

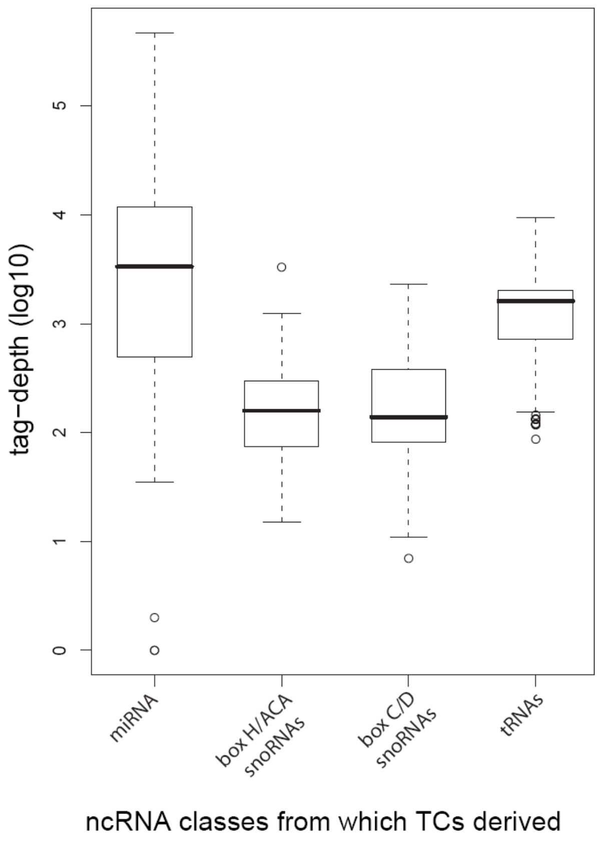

Figure S2. Tag-depth distributions of TCs covering full-length ncRNAs. MiRNA-derived TCs have a wide range of tag-depths, while the tag-depths of snoRNA-derived TCs are mostly between tens and thousands. TCs from tRNAs are with higher tag-depths than snoRNAs-derived TCs.

Figure S3

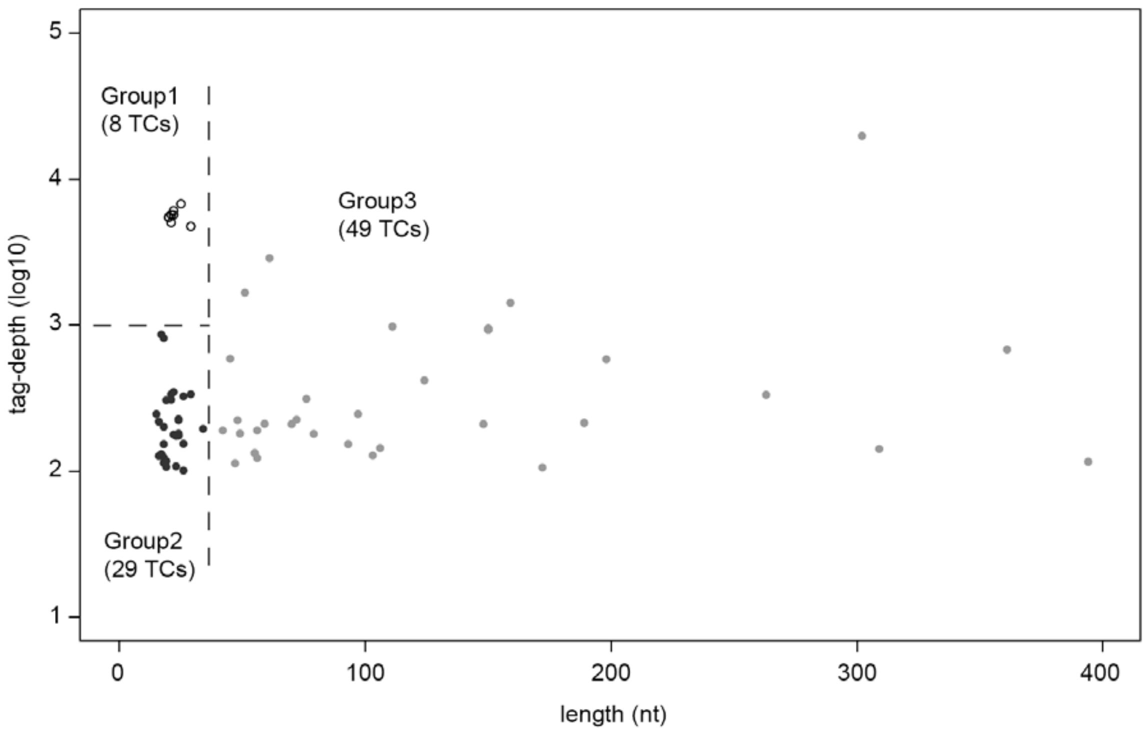

Figure S3. Length and tag-depth distribution of highly expressed unannotated TCs. Unannotated TCs with ambiguity in transcription direction are excluded (see text). TCs can be grouped as Group1: ≤40nt and tag-depth of ≥1000, Group2: ≤40nt and tag-depth of 100 ~ 999, and Group3: > 40nt.

Figure S4

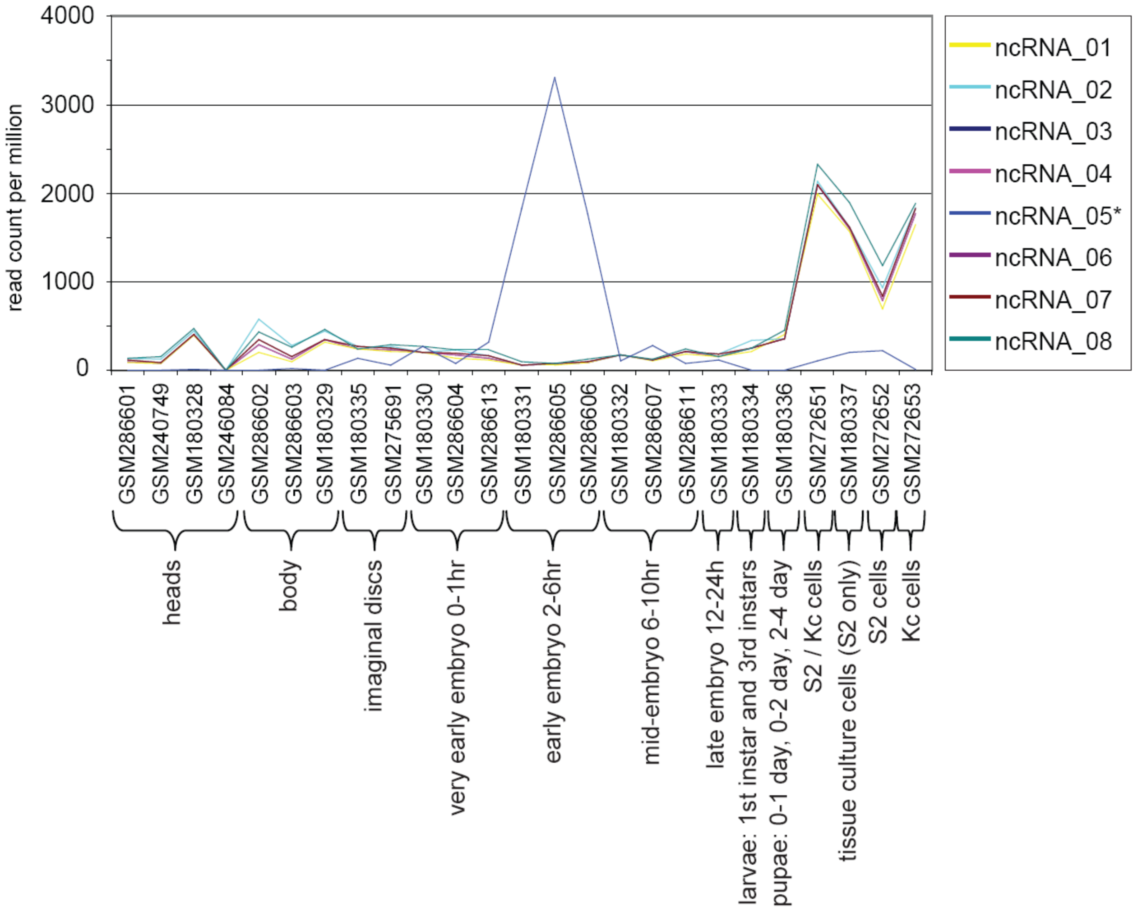

Figure S4. Number of sequence reads per million per different samples for Group1 TCs. All the 7 TCs sharing 18-mer motif (see the text) have high numbers of reads from S2 or Kc cells, while the vast majority of sequence reads for the tRNA-associated TC (ncRNA_05 : chr3X_3721726_3721755) is from early embryonic stages.

Figure S5

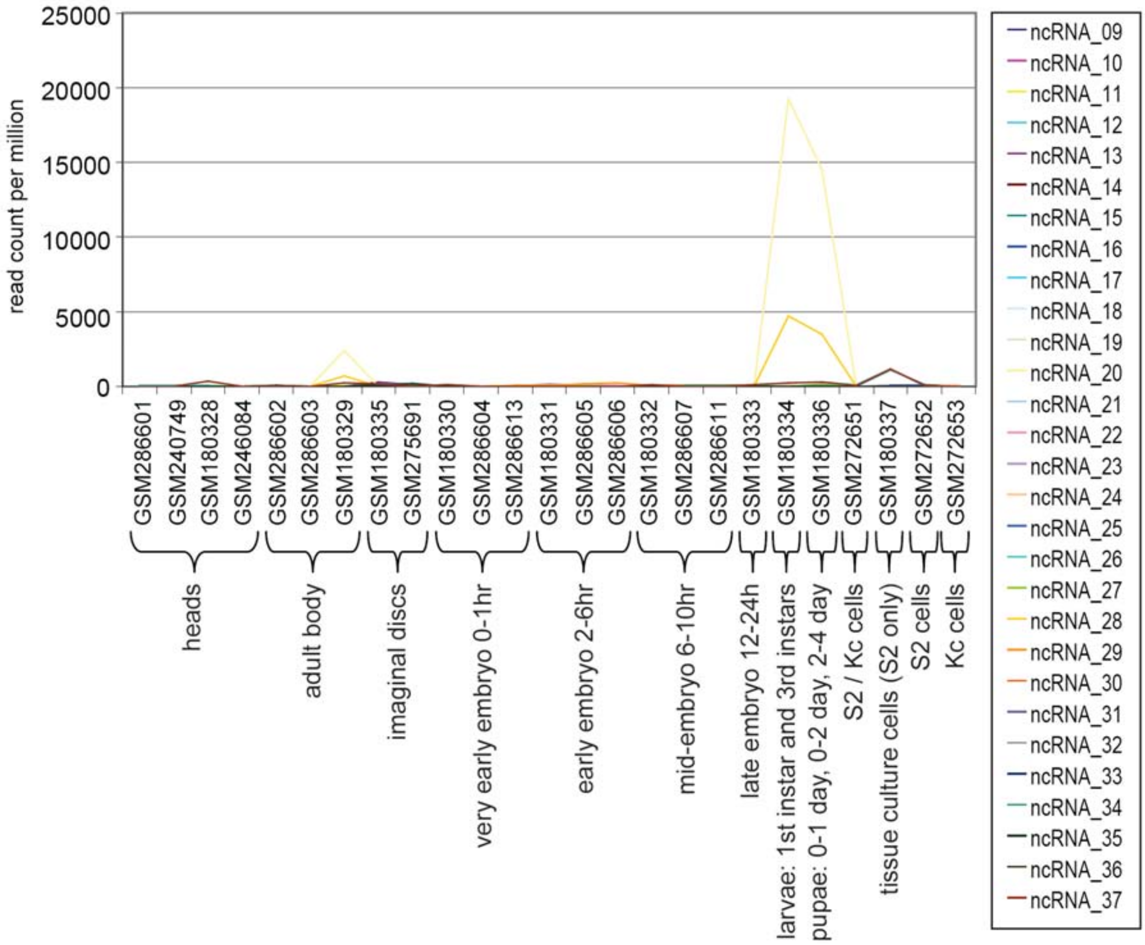

Figure S5. Number of sequence reads per million per different samples for Group2 TCs. While most of the TCs are relatively lowly expressed throughout different tissues, developmental stages and cultured cell lines, ncRNA_20 and ncRNA_28 have large number of sequence reads from adult body, larvae and pupae, and ncRNA_12, ncRNA_15 and ncRNA_37 are specifically expressed in S2 cells (GSM180337).

Figure S6

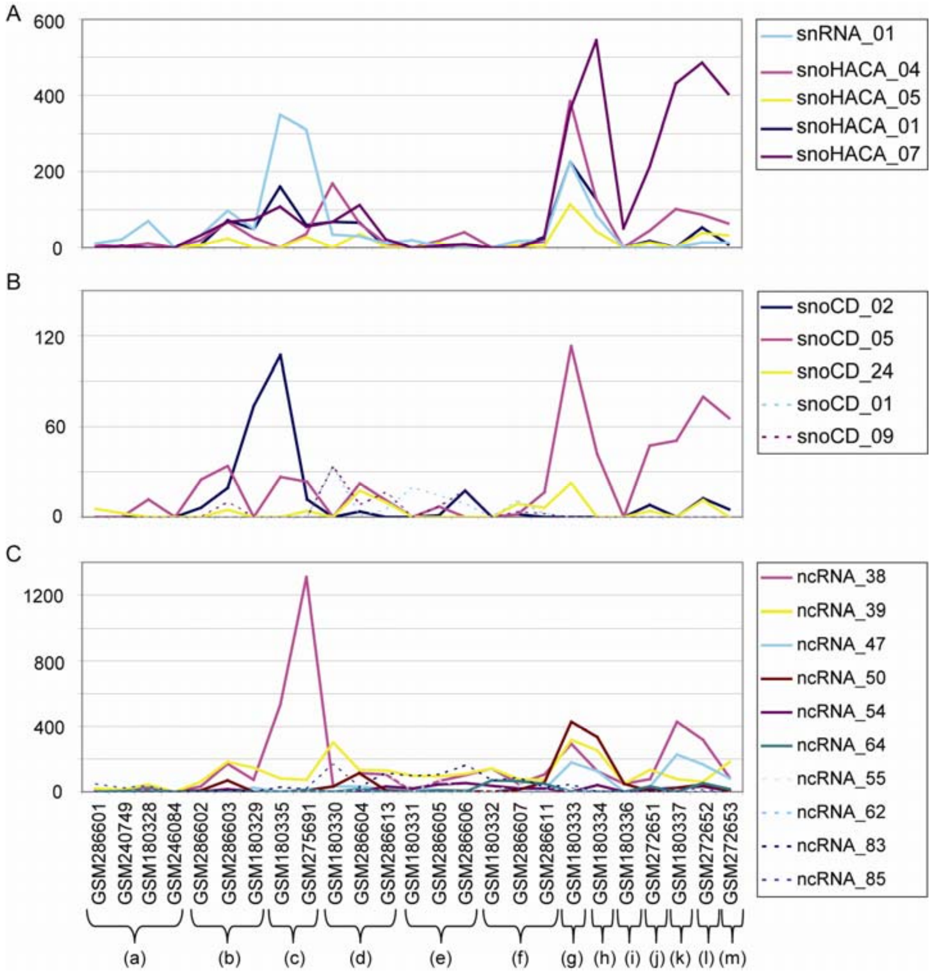

Figure S6. Number of sequence reads per million per sample for each of tested snRNA, snoRNA and unclassified ncRNA candidates. Relative sequence read numbers from each experiment for testes (A) snRNA and box H/ACA snoRNA candidates, (B) box C/D snoRNA candidates and (C) unclassified ncRNA candidates. Confirmed TCs are shown with solid lines and unconfirmed ones are shown with dashed lines. Overall, confirmed TCs have more number of sequence reads, especially from late embryos and S2 / Kc cells. TCs with negative results from Northern blot tend to have more sequence reads from early to mid embryonic stages. (a) heads, (b) adult body, (c) imaginal discs, (d) very early embryo (0-1), (e) early embryo (2-6), (f) mid embryo (6-10), (g) late embryo (12-24), (h) larvae: 1st instar and 3rd instars, (i) pupae: 0-1 day, 0-2 day, 2-4 day, (j) S2 and Kc cells, (k) tissue culture cells (S2 only), (l) S2 cells, (m) Kc cells.

Figure S7

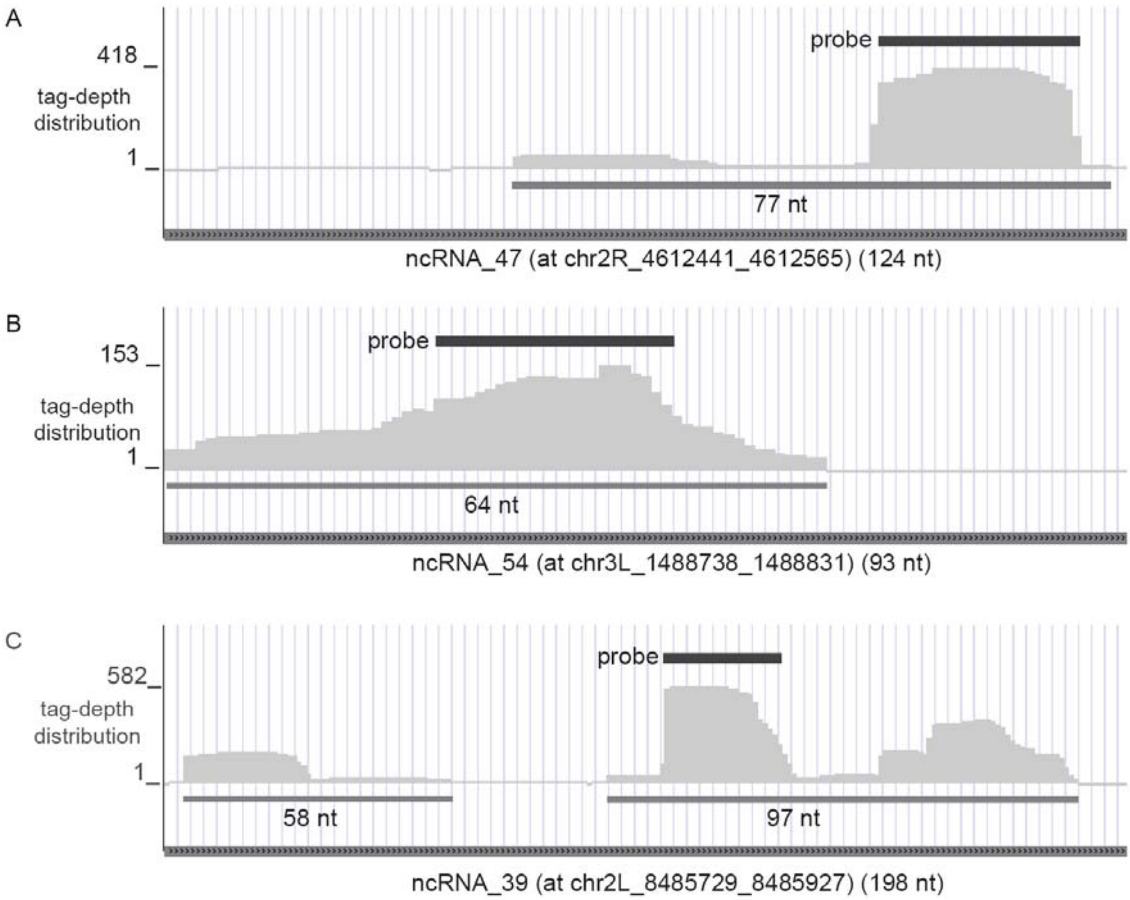

Figure S7. The accumulation of sequence reads per each base across the three TCs (A) ncRNA_47, (B) ncRNA_54 and (C) ncRNA_39. The tag-depth for position is shown in vertical light grey bars, and the horizontal thick and thin dark grey bars represent original TCs and processed TCs, respectively. In each case, the shorter processed TCs are in similar size with their corresponding Northern results.

Figure S8

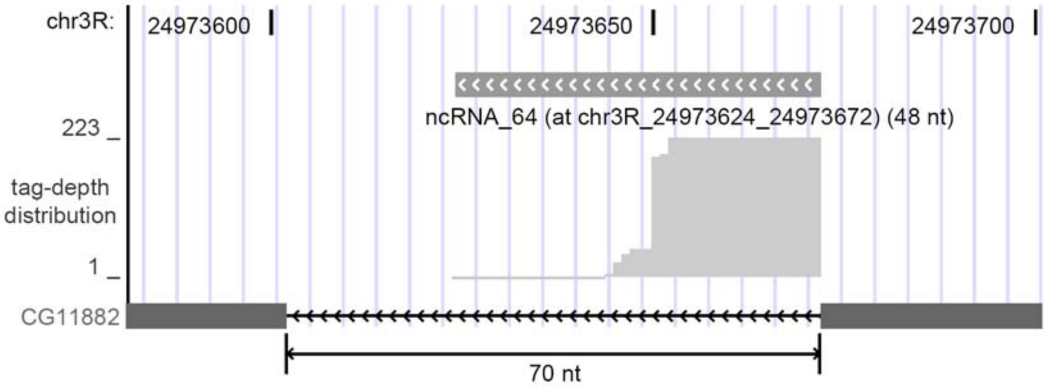

Figure S8. Screenshot of UCSC genome browser for an intronic TC chr3R_24973624_24973672. The depth of tags for each position is shown in vertical grey bars, and arrows stand for the transcriptional direction. While the length of the TC is 48 nt, the intron harbouring the TC is 70nt long, which is the same size to the Northern result of this TC.

Figures S9

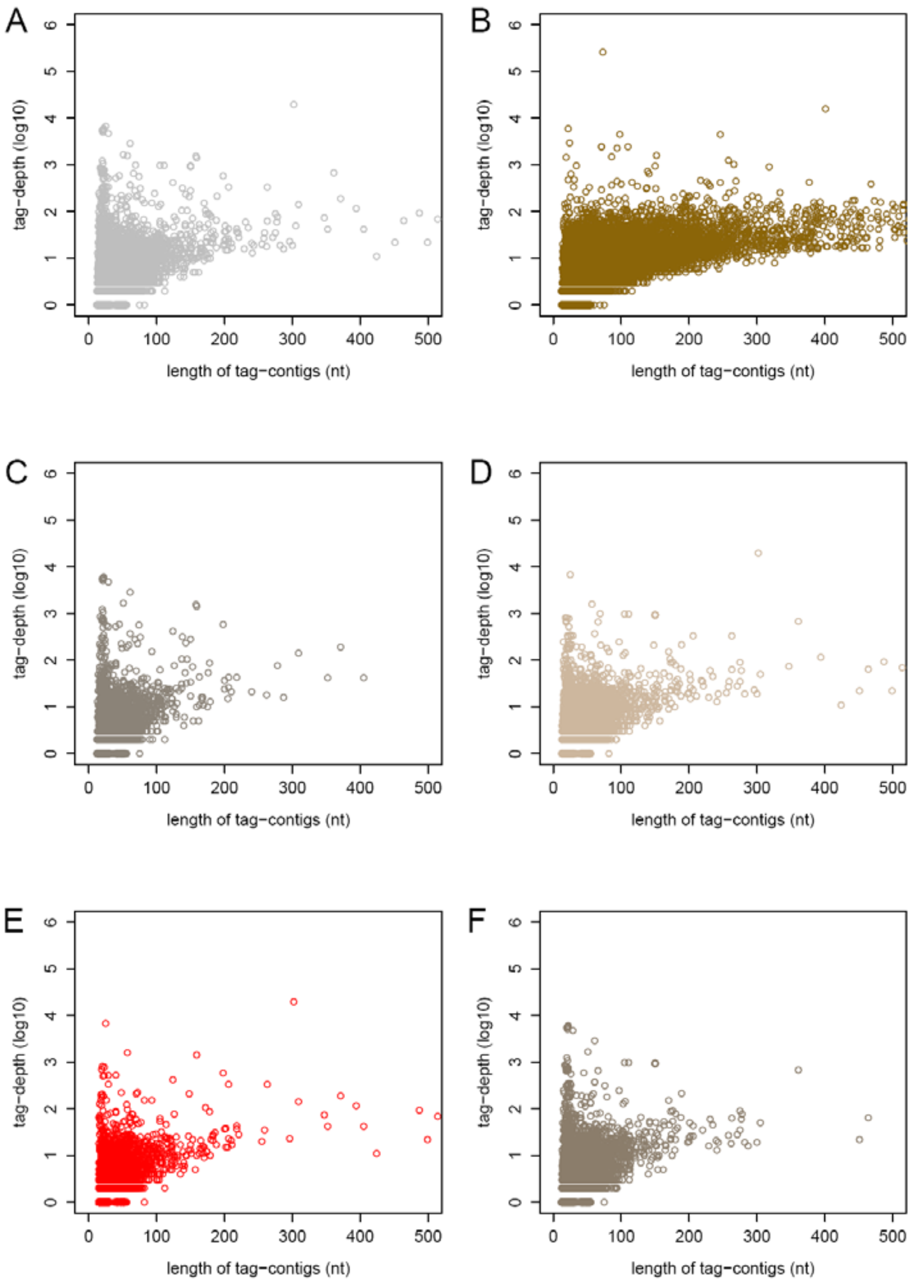

Figure S9. Length and tag-depth distributions of TCs of different categories. (A) All unannotated TCs. (B) TCs overlapping with exons. (C) Unannotated intronic TCs. (D) Unannotated TC in intergenic spacers. (E) Unannotated TCs containing evolutionary conserved sequence reads. (F) Unannotated TCs in non-conserved regions.

TABLE S1. Coverage of TCs over transposons and exons

	annotated	Overlapped by TCs	%
transposons*	24,191	15,703	64.9
exons	110,472	83,068	75.2

* Transposons were taken from FlyBase [1].

TABLE S2. Predictions of snoRNA and snRNA candidates

ID	locus	class	tag-depth	strand
snoCD_01	chr2L_6230_6304	C/D	27	+
snoCD_02	chr2L_232169_232250	C/D	42	-
snoCD_03	chr2L_901315_901392	C/D	9	-
snoCD_04	chr2L_1716521_1716588	C/D	7	-
snoCD_05*	chr2L_6917229_6917303	C/D	566	-
snoCD_06	chr2L_6926672_6926734	C/D	8	+
snoCD_07	chr2L_13216209_13216278	C/D	8	+
snoCD_08	chr2L_20749607_20749671	C/D	7	+
snoCD_09	chr2LHet_40314_40394	C/D	27	-
snoCD_10	chr2R_509417_509477	C/D	6	-
snoCD_11	chr2R_1499718_1499788	C/D	8	-
snoCD_12	chr2R_5454688_5454767	C/D	9	-
snoCD_13	chr2R_6391497_6391557	C/D	7	-
snoCD_14	chr2R_21138128_21138209	C/D	10	-
snoCD_15	chr2RHet_1922238_1922314	C/D	23	+
snoCD_16	chr3L_16365804_16365884	C/D	9	-
snoCD_17	chr3L_21168188_21168276	C/D	10	+
AJ784386	*chr3LHet_2398490_2398558*	*C/D*	*281*	+
snoCD_18	chr3R_1828621_1828718	C/D	8	-
snoCD_19	chr3R_12126308_12126370	C/D	8	+
snoCD_20	chr3R_27422603_27422676	C/D	11	-
snoCD_21	chr3R_27891609_27891681	C/D	6	-
snoCD_22	chr3RHet_2084563_2084659	C/D	9	-
snoCD_23	chr4_735935_736024	C/D	9	+
snoCD_24	chr4_86202_86271	C/D	993	-
snoCD_25	chrX_16333041_16333114	C/D	9	-
snoCD_26	chrX_18444686_18444748	C/D	20	+
snoHACA_01	chr2L_12432799_12432944	H/ACA	105	-
snoHACA_02	chr2L_12433035_12433185	H/ACA	34	-
AJ809564	*chr2L_22428188_22428326*	*H/ACA*	*317*	-
snoHACA_03	chr2R_6424004_6424157	H/ACA	72	-
snoHACA_04	chr2R_12744920_12745063	H/ACA	234	+
snoHACA_05	chr2R_15301861_15301998	H/ACA	106	-
snoHACA_06	chr3R_16738443_16738601	H/ACA	1555	-
snoHACA_07	chrX_915376_915513	H/ACA	25	-
snRNA_01**	chr3R_1020733_1020883	U4atac	282	-

* High similarity to snord53 (GenBank:X96652.1)

** High similarity to snRNA in *D.simulans* (NCBI Reference Seqquence:XR_050942.1)

TABLE S3. 18mer motif sites derived from group1 TCs with 0, 1 and 2 mismatches.

locus	strand	mismatches	gene	
chr2L_1856598_1856616	-	0	CG31665	exon
chr2R_4733785_4733803	+	0	*sns*	intron
chr2R_9632217_9632235	-	0	*fas*	intron
chr2R_13693471_13693489	-	0	*grh*	intron
chr2R_19535103_19535121	-	0	*retn*	intron
chrX_11524387_11524405	+	0	Ptp10D	Intron
chrX_12399633_12399651	-	0	CG2556	Intron
chrX_19880360_19880378	+	0	n/a	Intergenic
chr2L_3802018_3802036	-	1	CG3921	exon
chr2L_5356536_5356554	-	1	*nompC*	intron
chr2R_1419141_1419159	-	1	CG30438	intron
chr2R_2077630_2077648*	-	1	*Ptr*	intron
chr3R_2624832_2624850	+	1	*Dfd*	intron
chrX_4167694_4167712	-	1	n/a	Intergenic
chrX_11299498_11299516	-	1	*dlg1*	Intron
chrX_20283322_20283340	+	1	n/a	Intergenic
chrX_20495262_20495280	+	1	n/a	Intergenic
chr2L_1522186_1522204	-	2	n/a	Intergenic
chr2L_3808053_3808071	+	2	*l(2)k16918*	exon
chr2R_2040104_2040122*	-	2	*EcR*	Intron
chr2R_2040749_2040767*	+	2	*EcR*	Intron
chr2R_2049524_2049542*	-	2	*EcR*	Intron
chr2R_2049760_2049778*	+	2	*EcR*	Intron
chr2R_2050082_2050100*	+	2	*EcR*	Intron
chr2R_2077935_2077953*	+	2	*Ptr*	Intron
chr2R_20261644_20261662*	+	2	*bs*	Intron
chr3R_3965251_3965269*	-	2	n/a	Intergenic
chr3R_8974437_8974455	-	2	*timeout*	Intron
chr3R_10358310_10358328	+	2	n/a	Intergenic
chr3R_23984874_23984892	+	2	CD34354	Intron
chrX_16655710_16655728	-	2	*if*	Intron

Motif sites with higher number of mismatches include those with lower number of mismatches.

* Overlapping with 3' end of tRNA:N5

TABLE S4. Group3 TCs mapped to existing ncRNAs.

ID	locus	strand	tag-depth	
ncRNA_48	chr2R_7292203_7292273	+	210	tRNA
ncRNA_49	chr2R_7292691_7292839	-	210	tRNA
ncRNA_60	chr3R_2645849_2646151	-	19686	7SL RNA
ncRNA_65	chrX_4815890_4816040	-	931	siRNA cluster
ncRNA_66	chrX_4816173_4816323	-	931	siRNA cluster
ncRNA_67	chrX_4816456_4816606	-	931	siRNA cluster
ncRNA_68	chrX_4816739_4816889	-	931	siRNA cluster
ncRNA_69	chrX_4817009_4817159	-	944	siRNA cluster
ncRNA_70	chrX_4817279_4817429	-	931	siRNA cluster
ncRNA_71	chrX_4817562_4817712	-	931	siRNA cluster
ncRNA_72	chrX_4817845_4817995	-	931	siRNA cluster
ncRNA_73	chrX_4818128_4818278	-	931	siRNA cluster
ncRNA_74	chrX_4818411_4818561	-	931	siRNA cluster
ncRNA_75	chrX_4818694_4818844	-	931	siRNA cluster
ncRNA_76	chrX_4818977_4819127	-	931	siRNA cluster
ncRNA_77	chrX_4819260_4819410	-	944	siRNA cluster
ncRNA_78	chrX_4819530_4819680	-	931	siRNA cluster
ncRNA_79	chrX_4819800_4819950	-	944	siRNA cluster
ncRNA_80	chrX_4820070_4820220	-	944	siRNA cluster
ncRNA_81	chrX_4820340_4820490	-	944	siRNA cluster

TABLE S5. 29 unannotated TCs in group3.

ID	locus	tag-depth	strand		% of conserved reads
ncRNA_64	chr3R_24973624_24973672	223	-	Intronic	99.6
ncRNA_47	chr2R_4612441_4612565	418	+	Intronic	98.6
ncRNA_38	chr2L_3046745_3046904	1418	+	Intronic	35.9
ncRNA_54	chr3L_1488738_1488831	153	+	Intronic	21.1
ncRNA_85	chrX_21169300_21169372	225	-	Intronic	7.0
ncRNA_39	chr2L_8485729_8485927	582	+	Intronic	5.2
ncRNA_43	chr2R_1610220_1610281	2869	-	Intronic	0
ncRNA_51	chr2R_12095088_12095139	1667	-	Intronic	0
ncRNA_41	chr2L_21584617_21584662	587	-	Intronic	0
ncRNA_40	chr2L_21584031_21584107	312	-	Intronic	0
ncRNA_82	chrX_5726183_5726242	211	-	Intronic	0
ncRNA_63	chr3R_17624335_17624391	190	-	Intronic	0
ncRNA_53	chr3L_891424_891473	181	+	Intronic	0
ncRNA_52	chr3L_259971_260280	142	-	Intronic	0
ncRNA_62	chr3R_17142447_17142503	123	+	Intergenic	99.2
ncRNA_50	chr2R_9445408_9445580	106	-	Intergenic	48.1
ncRNA_83	chrX_12665793_12666187	116	+	Intergenic	22.8
ncRNA_55	chr3LHet_993964_994043	180	+	Intergenic	5.7
ncRNA_56	chr3LHet_994057_994168	976	+	Intergenic	0
ncRNA_58	chr3LHet_997026_997387	679	+	Intergenic	0
ncRNA_59	chr3LHet_997863_998126	333	+	Intergenic	0
ncRNA_57	chr3LHet_995772_995869	246	+	Intergenic	0
ncRNA_61	chr3R_9914042_9914231	214	+	Intergenic	0
ncRNA_84	chrX_19445331_19445373	190	+	Intergenic	0
ncRNA_86	chrX_21766140_21766246	144	+	Intergenic	0
ncRNA_44	chr2R_2293647_2293702	133	-	Intergenic	0
ncRNA_45	chr2R_2295817_2295872	133	-	Intergenic	0
ncRNA_46	chr2R_2304645_2304748	128	-	Intergenic	0
ncRNA_42	chr2R_173296_173343	113	-	Intergenic	0

TABLE S6. Conservation of sequence tags comprising ncRNA-derived TCs

	Overlapping TCs	Total reads	Conserved reads*	%
miRNA	153	64,565	59,221	91.7
Box H/ACA snoRNAs	109	19,340	13,210	68.3
Box C/D snoRNAs	107	28,750	20,967	72.9
tRNAs	295	589,630	568,037	96.3
snRNAs	29	54,531	53,270	97.7
snmRNAs**	17	2,701	2,069	76.6
Other ncRNAs	205	83,992	76,956	91.6

*Sequence reads that have at least 15bp in phastCons elements [2].
**20 snmRNAs that are associated with *His* gene cluster were excluded for their repetitive nature.

References

1. Drysdale R, the FlyBase C: **FlyBase**. In: *Drosophila*. 2008: 45-59.
2. Siepel A, Bejerano G, Pedersen JS, Hinrichs AS, Hou M, Rosenbloom K, Clawson H, Spieth J, Hillier LW, Richards S *et al*: **Evolutionarily conserved elements in vertebrate, insect, worm, and yeast genomes**. *Genome Res* 2005, **15**:1034-1050.
