# Supplementary material for: Identification of novel non-coding RNAs using profiles of short sequence reads from next generation sequencing data
Source: BMC Genomics. 2010 Feb 1;11:77. doi: 10.1186/1471-2164-11-77 (PMC2825236; doi:10.1186/1471-2164-11-77)

Identification of novel non-coding RNAs using profiles of short sequence reads from next generation sequencing data

Jung et al.

Sequence conservation and structures of snoRNA predictions

Box H/ACA snoRNA candidates

snoHACA_01: chr2L_12432799_12432944 (validated)

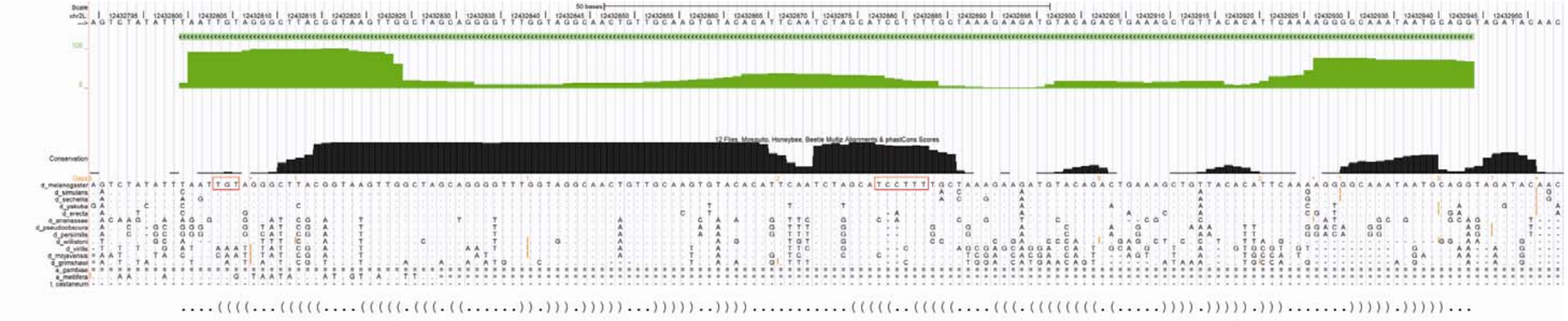

snoHACA_02: chr2L_12433035_12433185

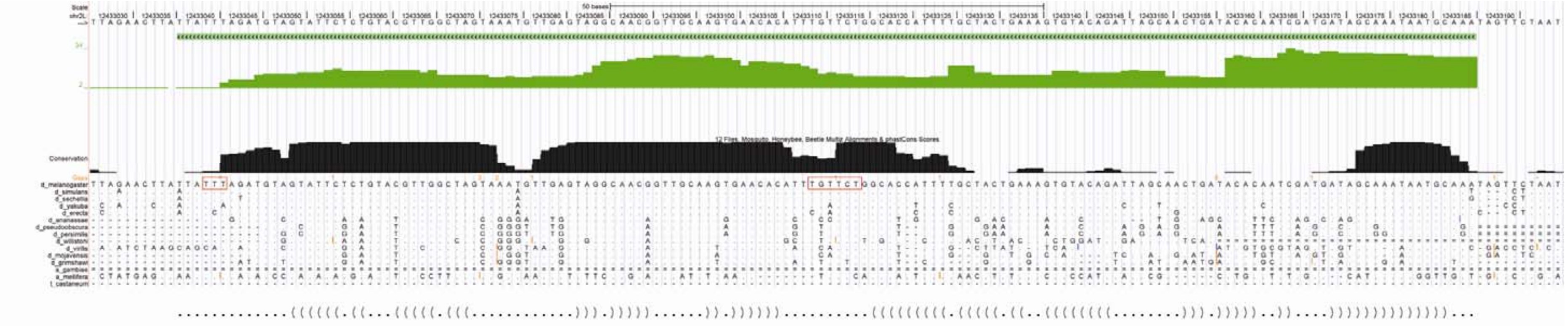

snoHACA_03: chr2R_6424004_6424157

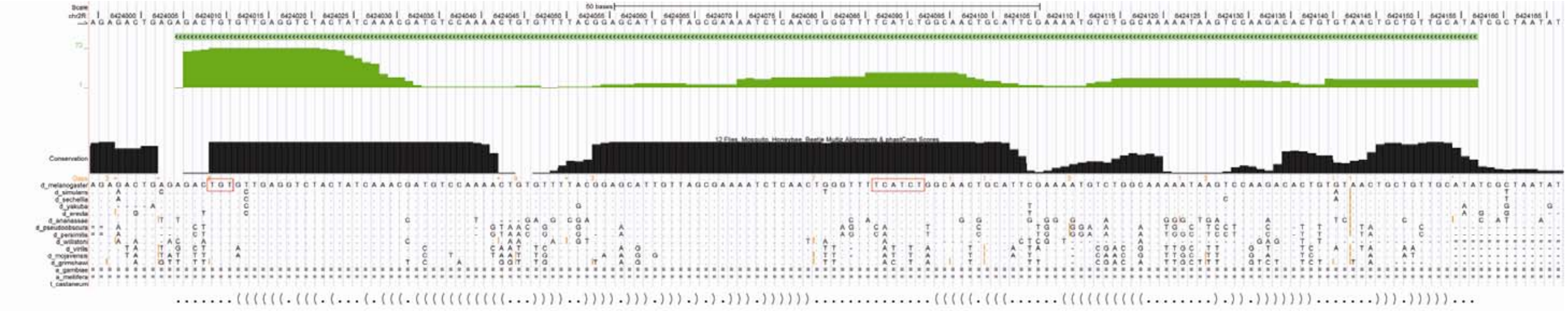

snoHACA_04: chr2R_12744920_12745063 (validated)

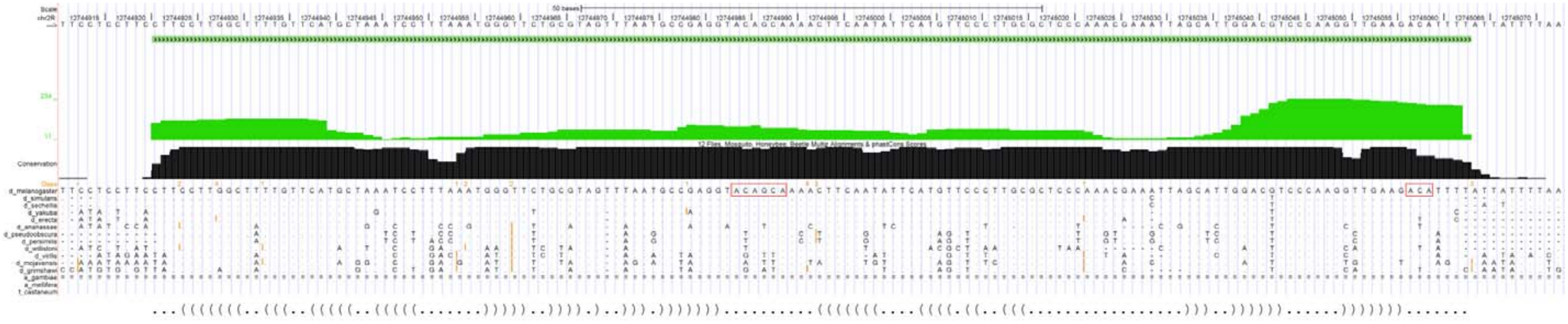

snoHACA_05: chr2R_15301861_15301998 (validated)

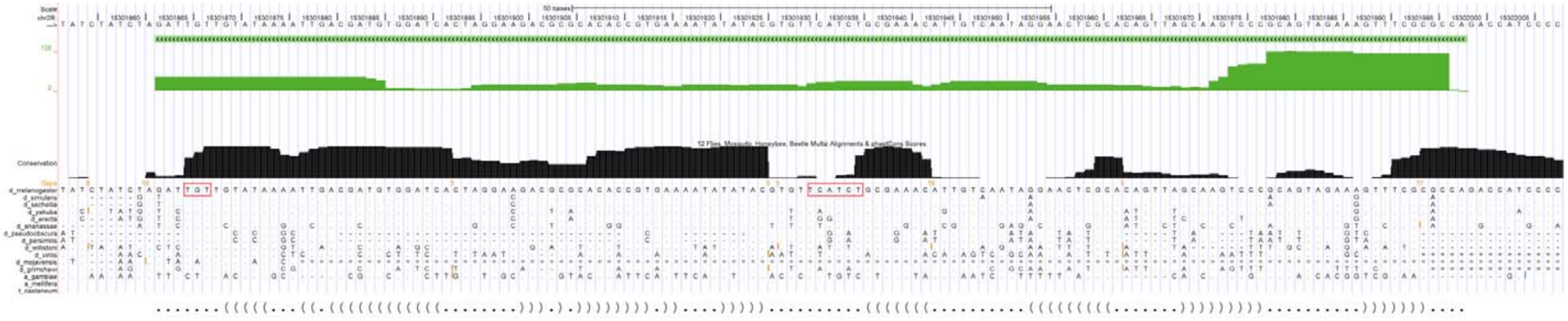

snoHACA_06: chr3R_16738443_16738601 (validated)

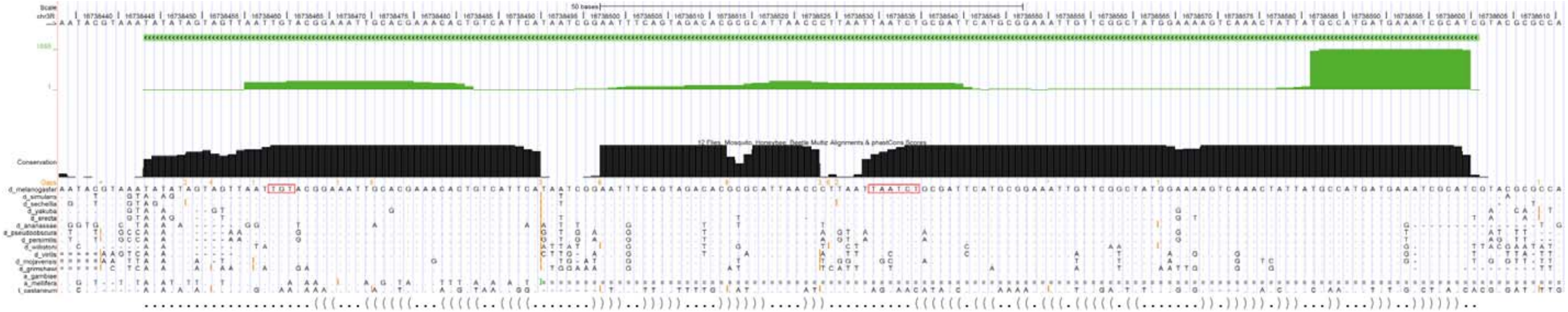

snoHACA_07: chrX_915376_915513

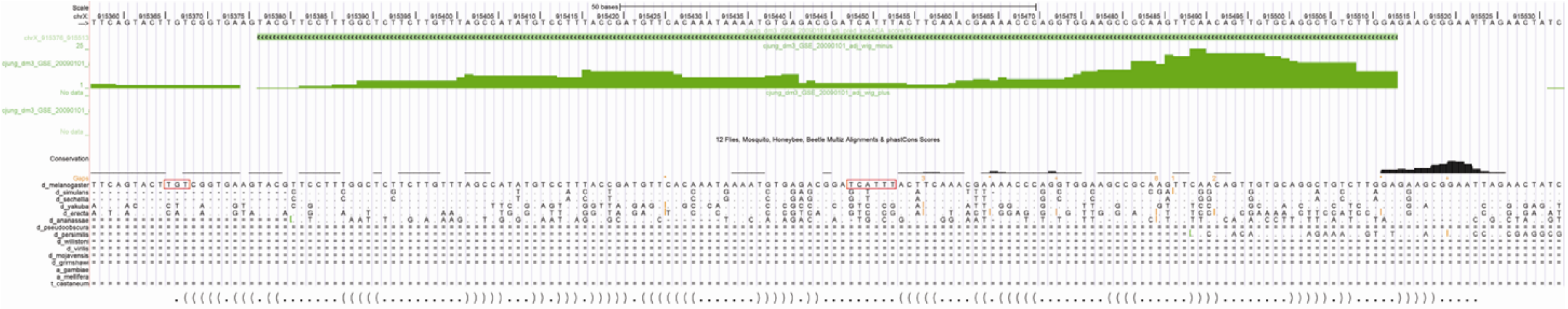

Box H/ACA snoRNA candidates

snoCD_01: chr2L_6230_6304

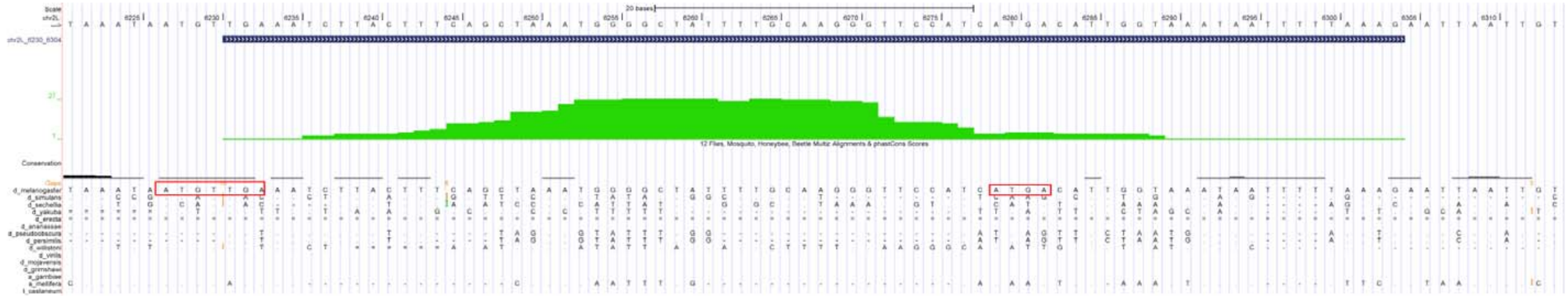

snoCD_02: chr2L_232169_232250 (validated)

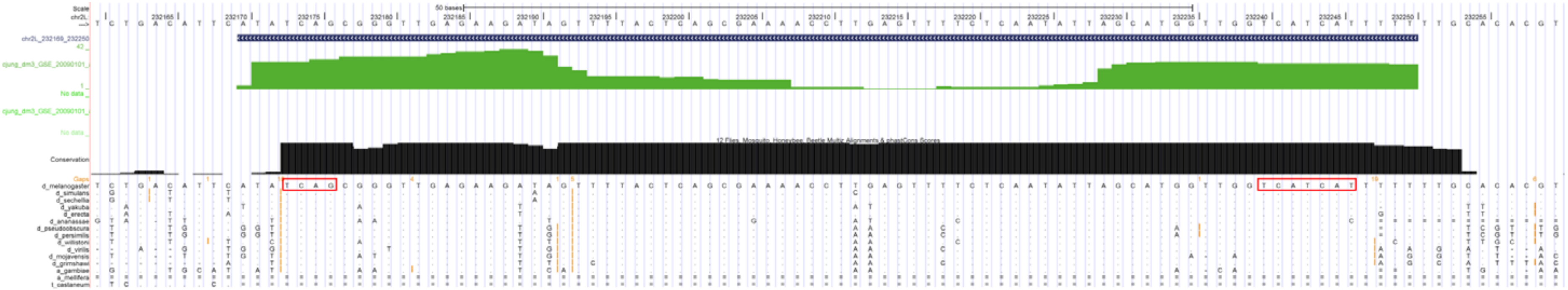

snoCD_03: chr2L_901315_901392

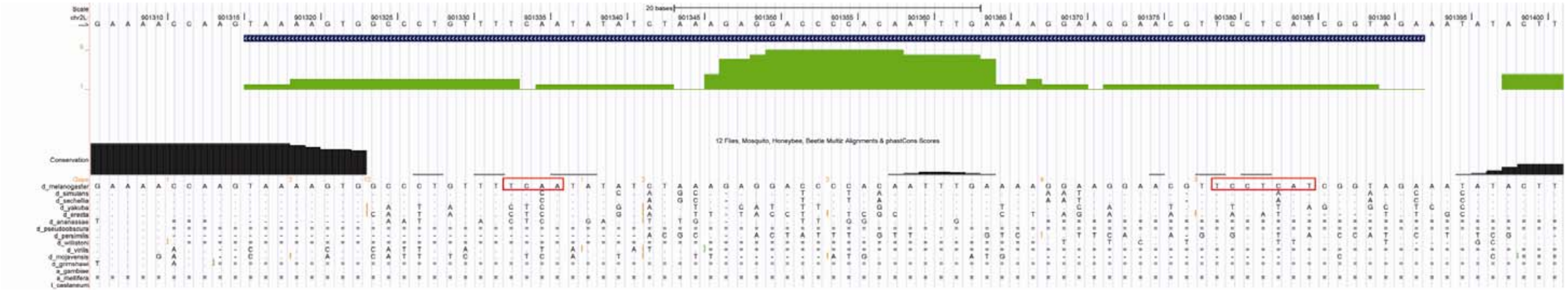

snoCD_04: chr2L_1716521_1716588

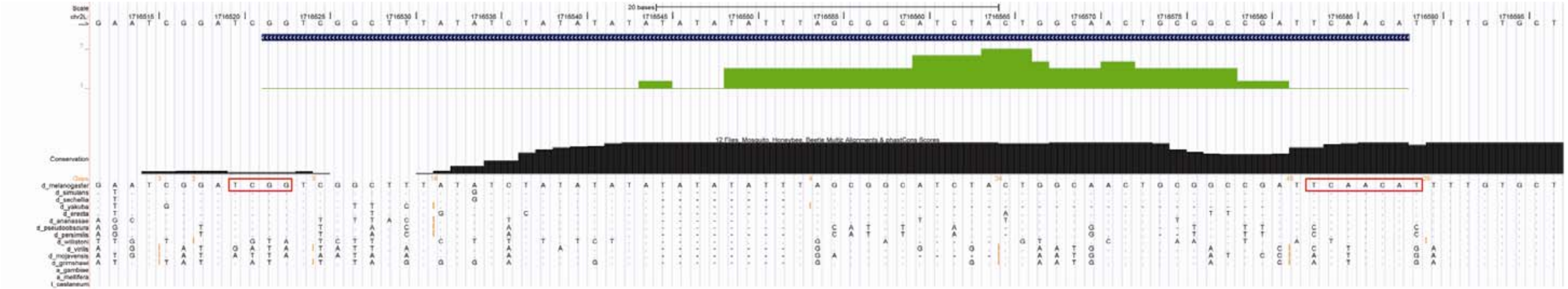

snoCD_05: chr2L_6917229_6917303 (validated)

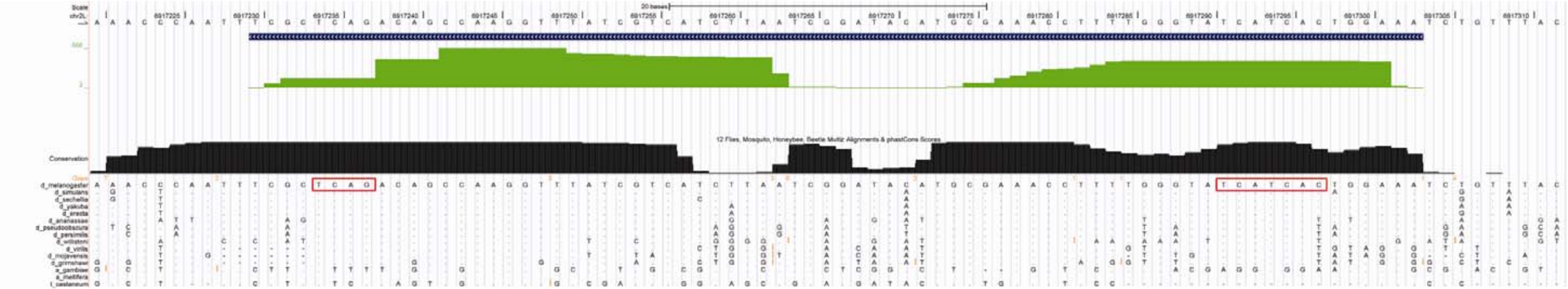

snoCD_06: chr2L_6926672_6926734

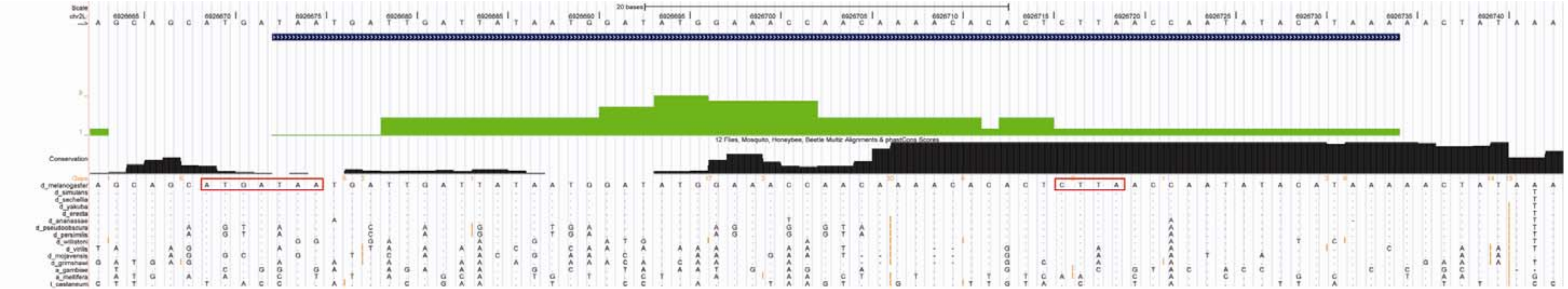

snoCD_07: chr2L_13216209_13216278

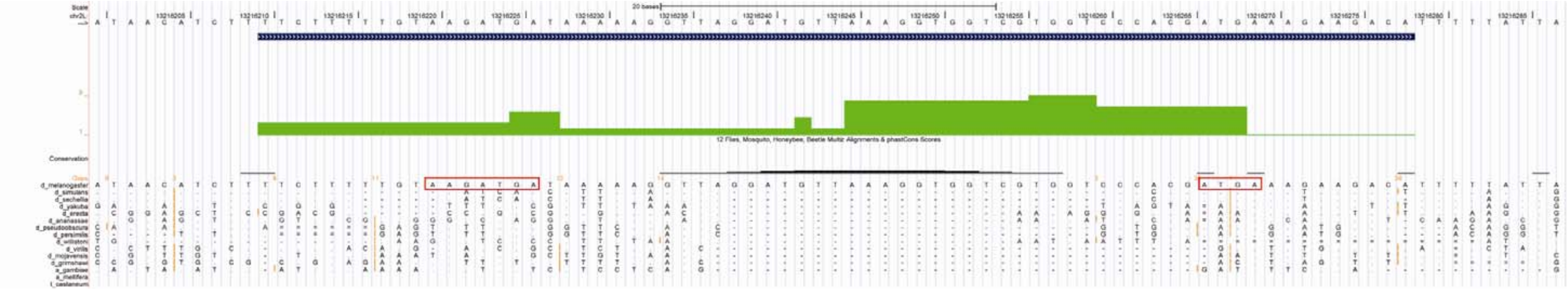

snoCD_08: chr2L_20749607_20749671

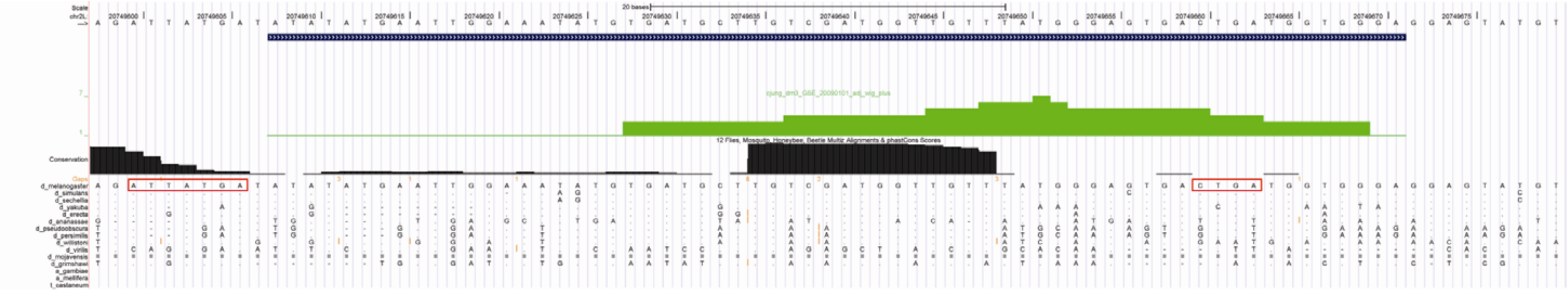

snoCD_09: chr2LHet_40314_40394

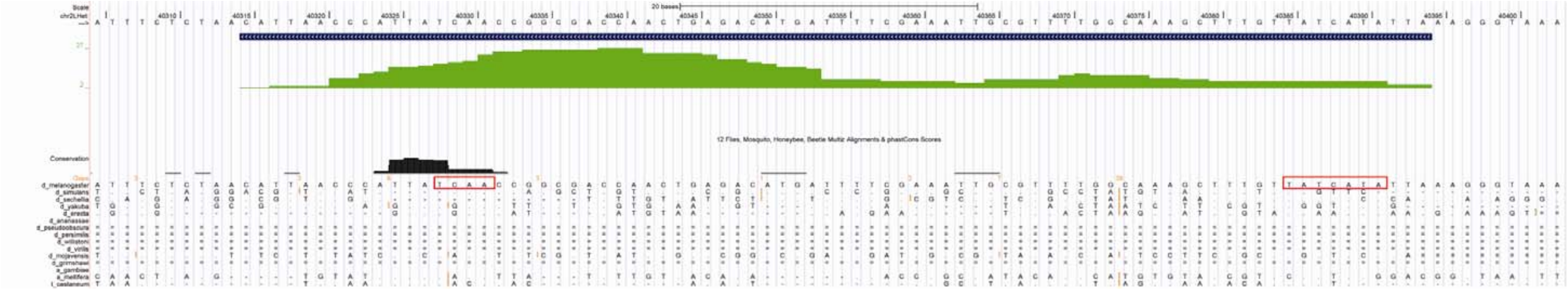

snoCD_10: chr2R_509417_509477

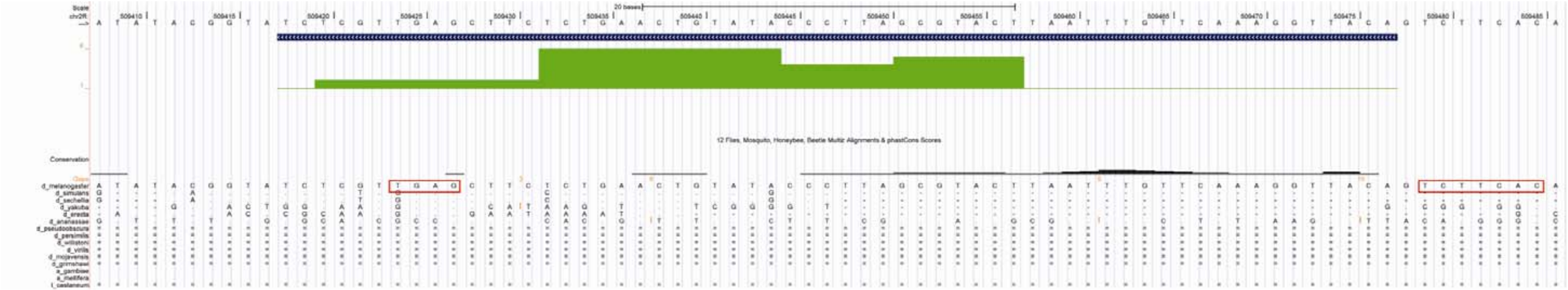

snoCD_11: chr2R_1499718_1499788

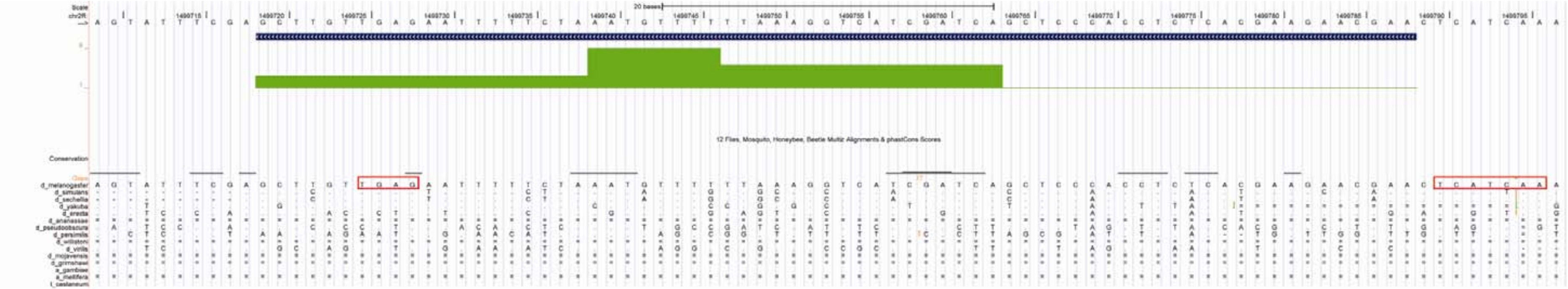

snoCD_12: chr2R_5454688_5454767

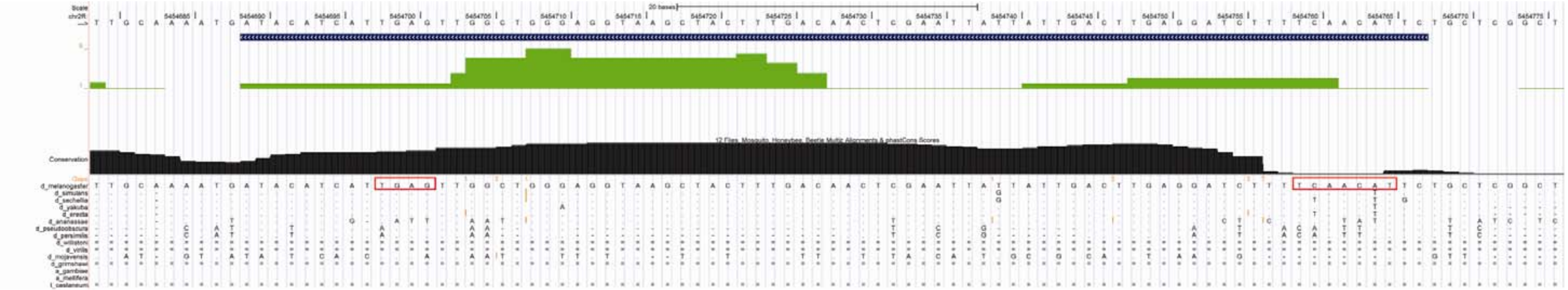

snoCD_13: chr2R_6391497_6391557

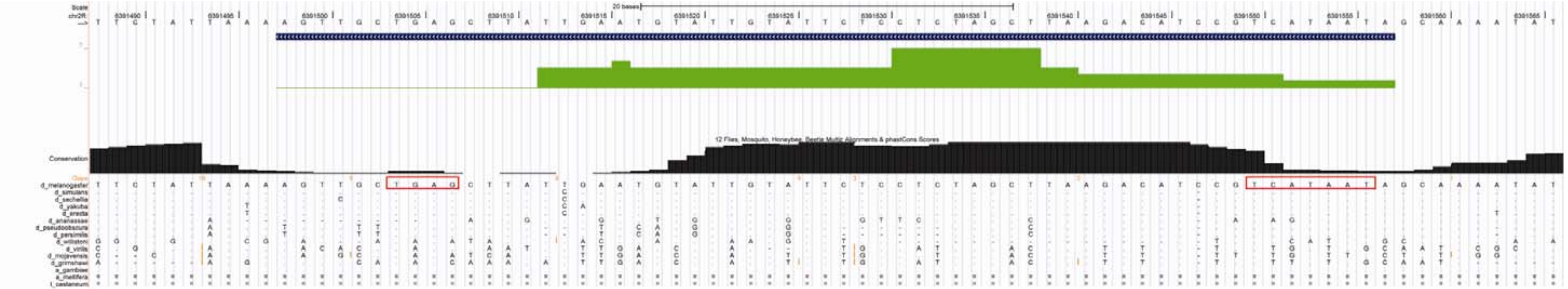

snoCD_14: chr2R_21138128_21138209

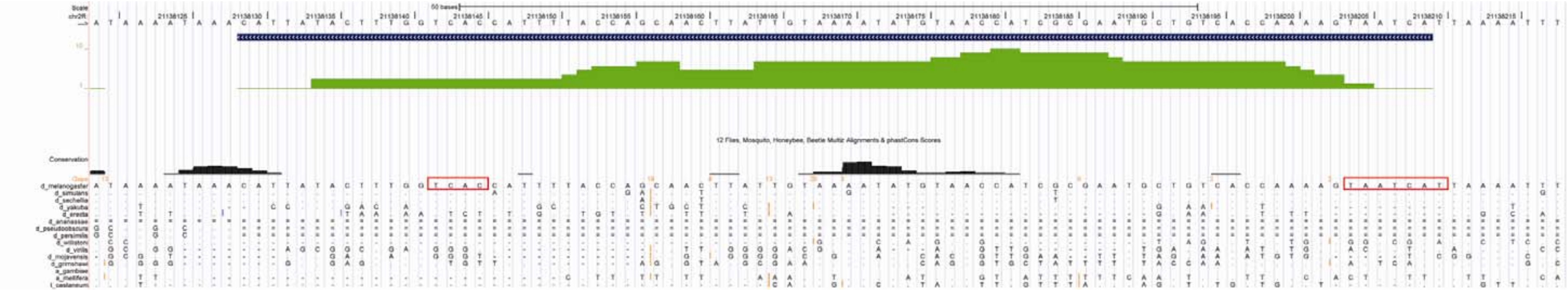

snoCD_15: chr2RHet_1922238_1922314

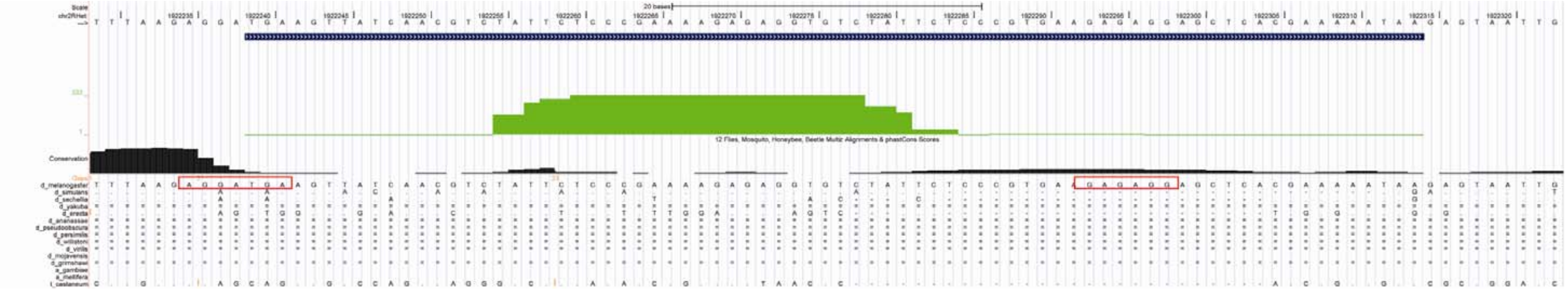

snoCD_16: chr3L_16365804_16365884

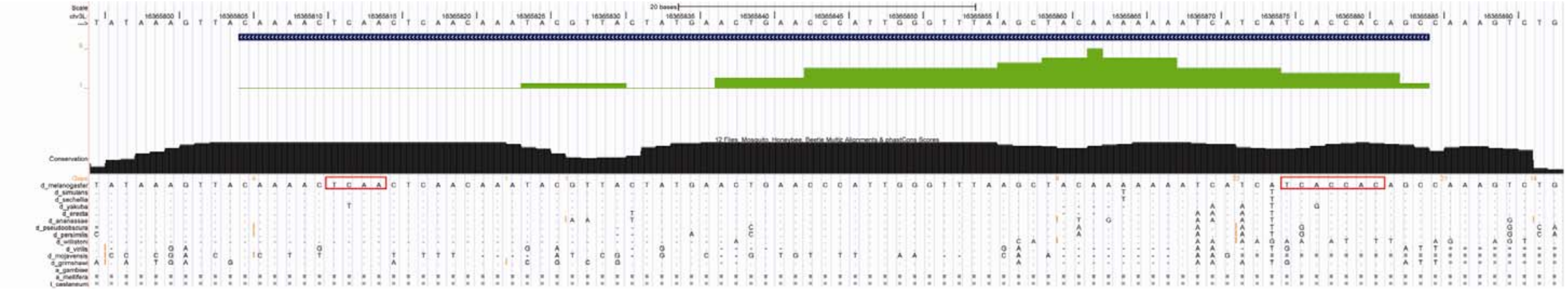

snoCD_17: chr3L_21168188_21168276

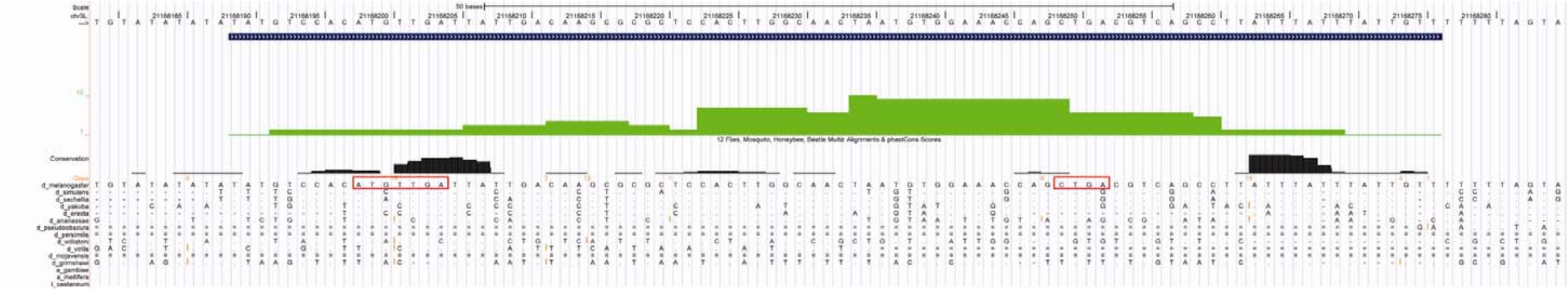

snoCD_18: chr3R_1828621_1828718

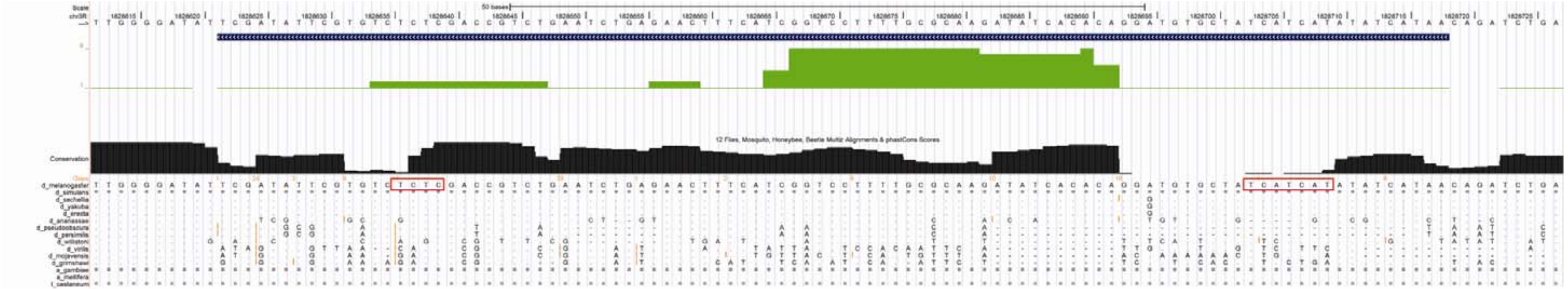

snoCD_19: chr3R_12126308_12126370

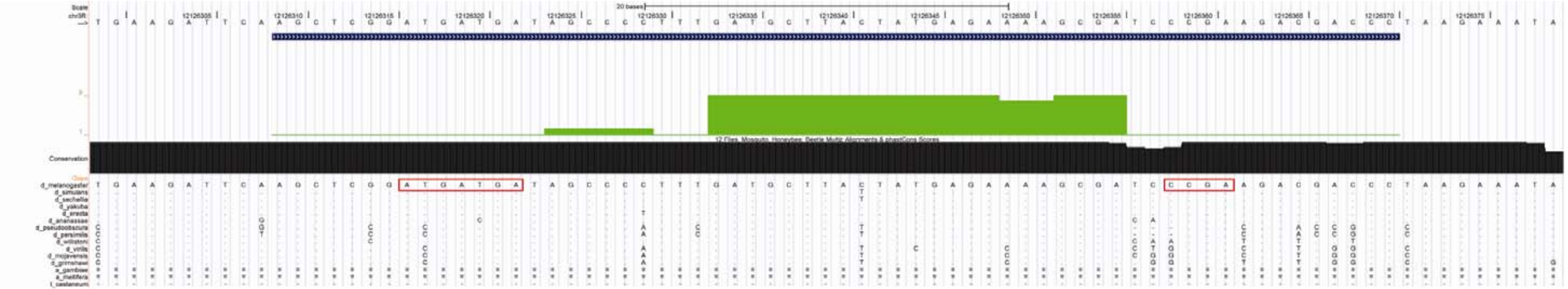

snoCD_20: chr3R_27422603_27422676

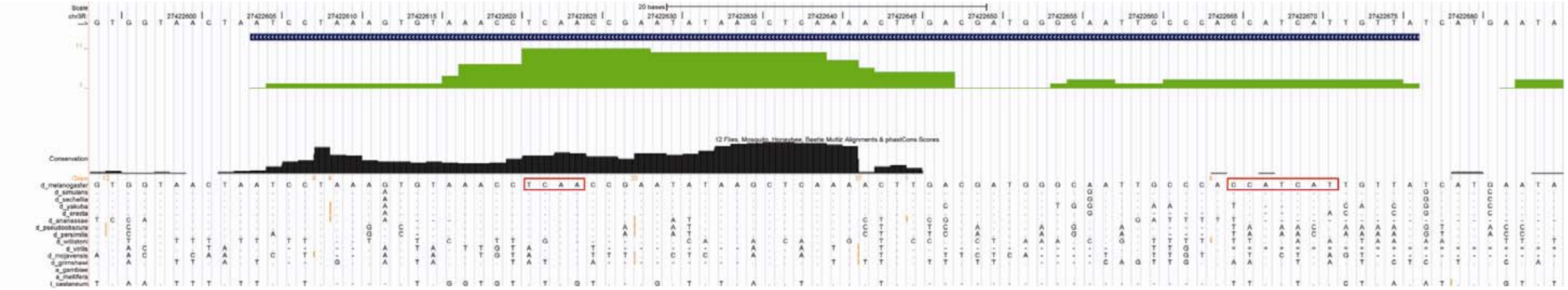

snoCD_21: chr3R_27891609_27891681

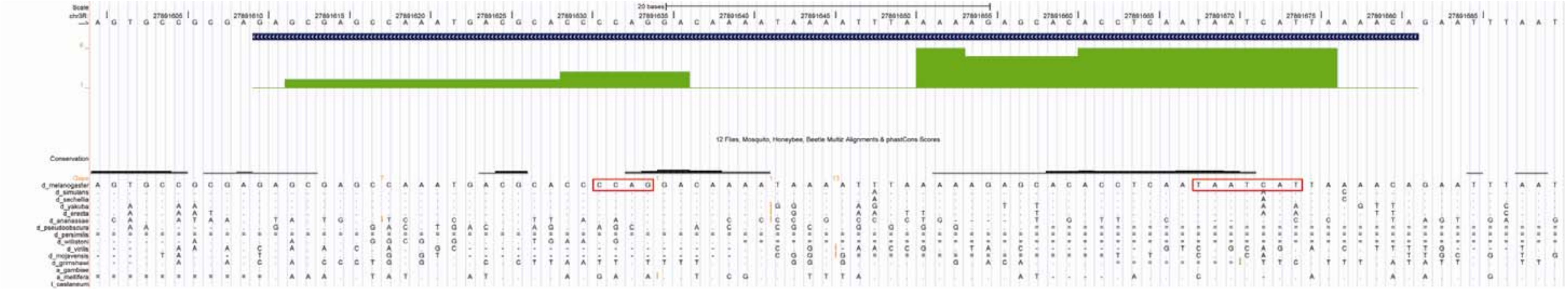

snoCD_22: chr3RHet_2084563_2084659

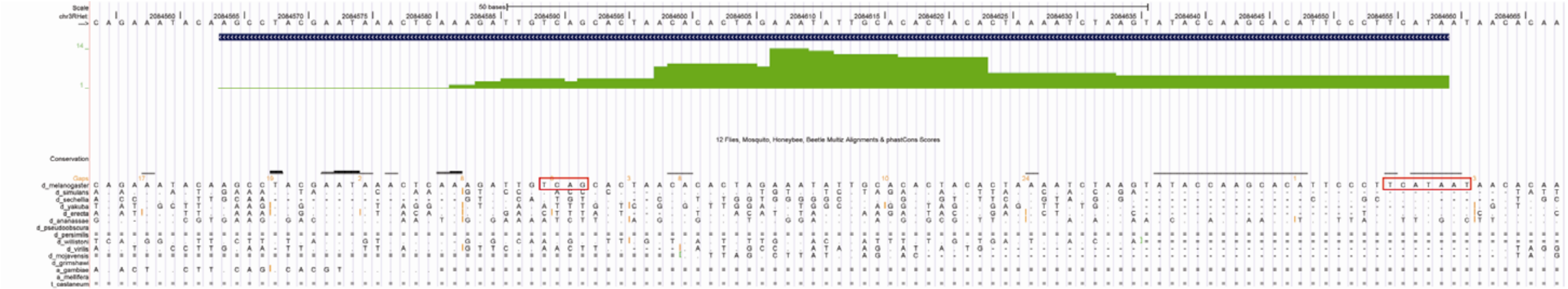

snoCD_23: chr4_735935_736024

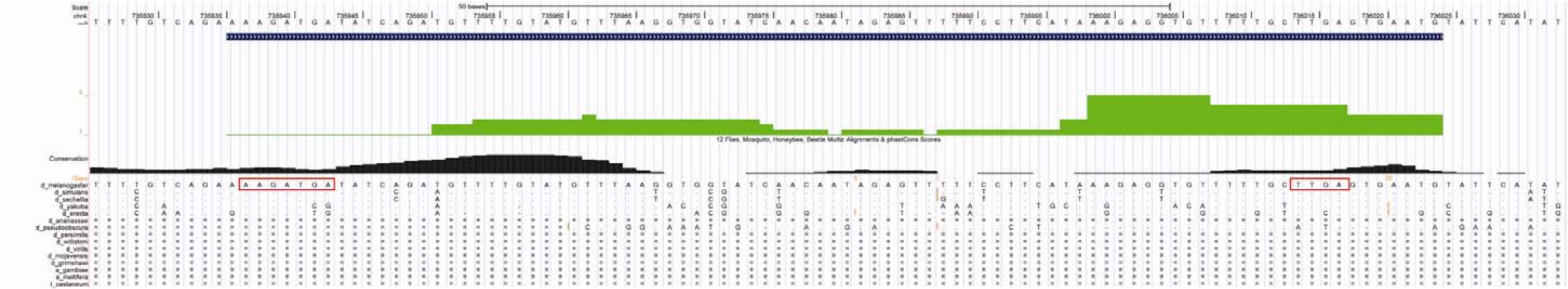

snoCD_24: chr4_86202_86271 (validated)

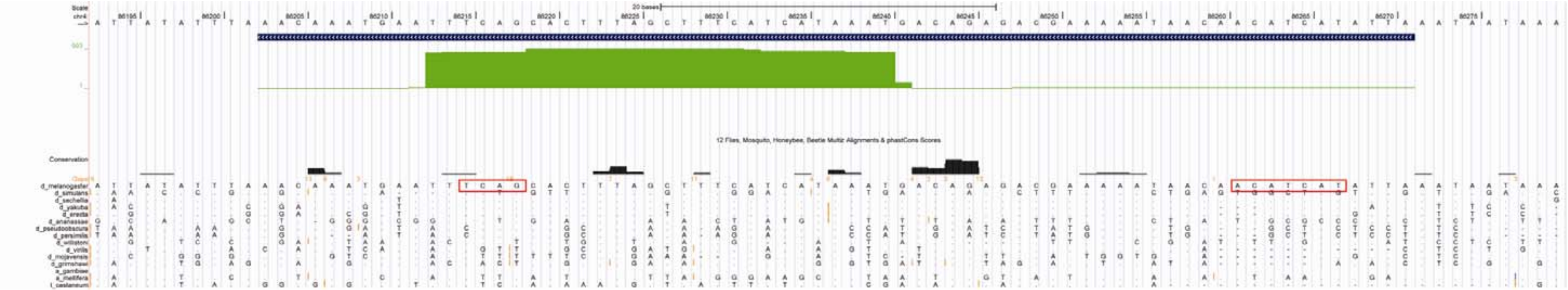

snoCD_25: chrX_16333041_16333114

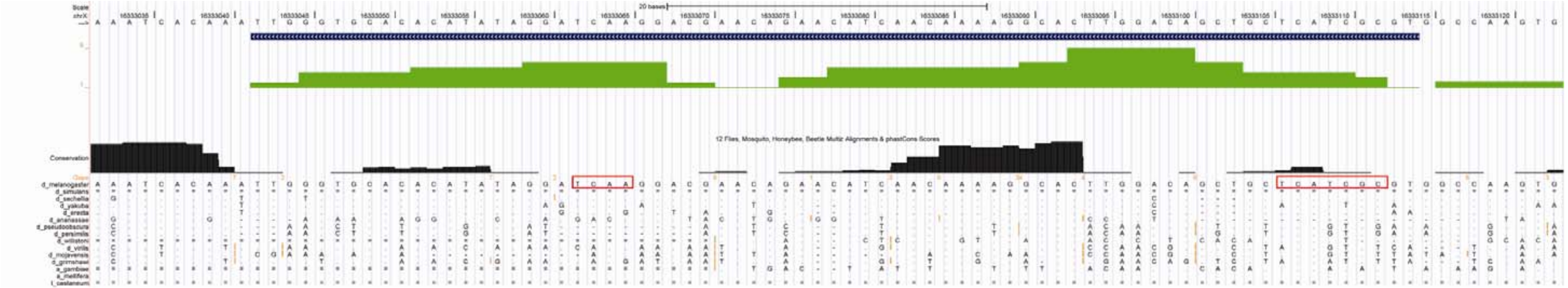

snoCD_26: chrX_18444686_18444748

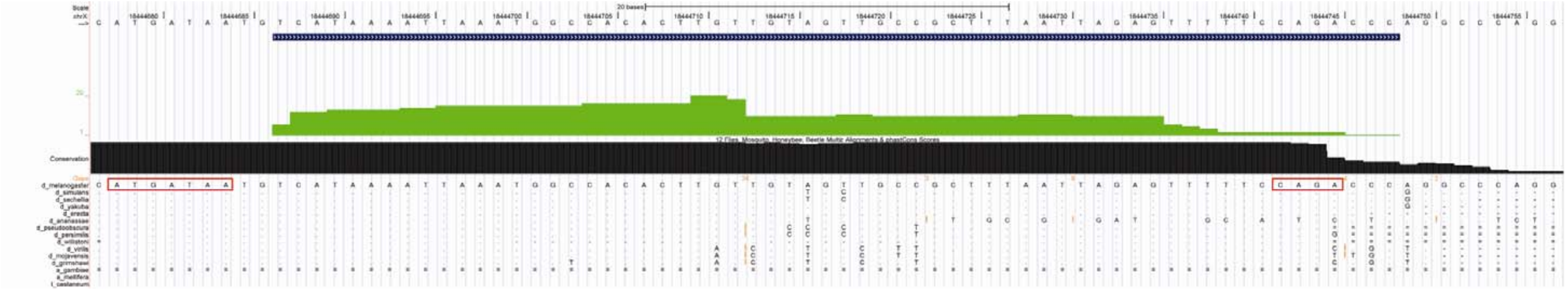

Supplement: Additional file 6 — Sequence conservation and secondary structure predictions of snoRNA candidates. This file contains UCSC genome browser screenshots of 7 box H/ACA snoRNA candidates and 26 box C/D snoRNA candidates showing sequence conservation and tag distributions over each candidate. The positions of box H, ACA, C and D motifs are indicated by the red boxes. Predicted secondary structures of box H/ACA snoRNA candidates are provided below each of the screenshots. [file 1471-2164-11-77-S6.PDF]
